# Evolution of transcriptional networks in yeast: alternative teams of transcriptional factors for different species

**DOI:** 10.1186/s12864-016-3102-7

**Published:** 2016-11-11

**Authors:** Adriana Muñoz, Daniella Santos Muñoz, Aleksey Zimin, James A. Yorke

**Affiliations:** 1 Institute for Physical Science and Technology, University of Maryland, College Park, Maryland, 20742 USA; 2 Department of Mathematics, University of Maryland, College Park, Maryland, 20742 USA; 3 Department of Physics, University of Maryland, College Park, Maryland, 20742 USA; 4 Cold Spring Harbor Laboratory, 1 Bungtown Rd., Cold Spring Harbor, 11724 NY USA; 5 Faculty of Sciences, University of Ottawa, Ottawa, K1N 6N5 ON Canada; 6 Faculty of Engineering, University of Ottawa, Ottawa, K1N 6N5 ON Canada

**Keywords:** Transcription factor, Rewiring, Evolution, Regulation, Transcriptional networks, Yeast, Ascomycota

## Abstract

**Background:**

The diversity in eukaryotic life reflects a diversity in regulatory pathways. Nocedal and Johnson argue that the rewiring of gene regulatory networks is a major force for the diversity of life, that changes in regulation can create new species.

**Results:**

We have created a method (based on our new “ping-pong algorithm) for detecting more complicated rewirings, where several transcription factors can substitute for one or more transcription factors in the regulation of a family of co-regulated genes. An example is illustrative. A rewiring has been reported by Hogues et al. that RAP1 in *Saccharomyces cerevisiae* substitutes for TBF1/CBF1 in *Candida albicans* for ribosomal RP genes. There one transcription factor substitutes for another on some collection of genes. Such a substitution is referred to as a “rewiring”. We agree with this finding of rewiring as far as it goes but the situation is more complicated. Many transcription factors can regulate a gene and our algorithm finds that in this example a “team” (or collection) of three transcription factors including RAP1 substitutes for TBF1 for 19 genes. The switch occurs for a branch of the phylogenetic tree containing 10 species (including *Saccharomyces cerevisiae*), while the remaining 13 species (*Candida albicans*) are regulated by TBF1.

**Conclusions:**

To gain insight into more general evolutionary mechanisms, we have created a mathematical algorithm that finds such general switching events and we prove that it converges. Of course any such computational discovery should be validated in the biological tests. For each branch of the phylogenetic tree and each gene module, our algorithm finds a sub-group of co-regulated genes and a team of transcription factors that substitutes for another team of transcription factors. In most cases the signal will be small but in some cases we find a strong signal of switching. We report our findings for 23 Ascomycota fungi species.

**Electronic supplementary material:**

The online version of this article (doi:10.1186/s12864-016-3102-7) contains supplementary material, which is available to authorized users.

## Background

**Fig. 1:**
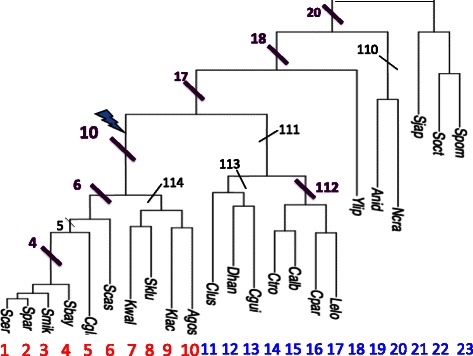
Tree phylogeny for 23 species of yeast. We test each of the 12 selected branches (marked as #4, #5, #6, #10, #114, etc.) to partition the species in the tree for rewiring events. Note that the partition numbers that are one or two digits indicates the branch includes all species up to that species number. A whole genome duplication is indicated in branch #10. Each branch partitions the set of species into two sets M (the species on that branch) and *M* ^⋆^ (the remaining species). The 23 species are: *Saccharomyces (S.) cerevisiae (1), S. paradoxus(2), S. mikatae (3), S. bayanus (4), Candida (C.) glabrata (5), S. castellii (6), Kluyveromyces (K.) waltii (7), S. kluyveri (8), K. lactis (9), Ashbya gossypii (10), Clavispora lusitaniae (11), Debaryomyces hansenii (12), C. guilliermondii (13), C. tropicalis (14), C. albicans (15), C. parapsilosis (16), Lodderomyces elongisporus (17), Yarrowia lipolytica (18), Aspergillus nidulans (19), Neurospora crassa (20), Schizosaccharomyces japonicus (21), Schizosaccharomyces octosporus (22), Schizosaccharomyces pombe (23)*

One of the several ways that species evolve and diverge from each other is through changes in regulatory networks and more specifically through changes in the regulation of genes by transcription factors. The 23 species with an established phylogeny in Fig. 1 are collectively an excellent environment or model for the study of gene regulation in general. To investigate evolutionary changes, we generally compare regulation in the species in one branch of the phylogenetic tree and compare that with the remaining species. A group of functionally linked and co-regulated genes is called a “regulon”. A regulon (and its function) may be preserved across a family of related species despite changes in regulation. In the review [1], Li and Johnson propose three different scenarios for the evolution of transcriptional networks in yeast. Their scenarios are (1) “transcription factor turnover” where the transcription factor is conserved (as well as the transcription factor binding probability), but membership of genes in the regulon can change; (2) “transcription factor rewiring” or “switching” where the regulon members are conserved, but the regulation switches from one transcription factor to another transcription factor; (3) evolution of combinatorial interactions between transcription factors due to direct protein-protein contacts between DNA binding proteins.

In this paper we are interested in Scenario 2. Hogues et al. [2] report an example of scenario (2) change in regulation, namely that in *Saccharomyces cerevisiae* the transcription factor RAP1 regulates ribosomal RP genes, while in the same conditions in *Candida albicans* the regulation of the same ribosomal RP genes is done by the transcription factor TBF1 (and sometimes also CBF1). There one transcription factor for certain species is replaced by another transcription factor for different species, carrying out the regulation of the same collection of genes. In order for a collection of related genes to preserve their function, we must expect change in transcription factors to be carried out for a collection of genes. Additional such cases have been documented for yeast genes involved in mating [3] and in galactose metabolism [4, 5]. See also cases discussed in [6] and references therein.

Scenario (2) can also be discussed in terms of “motifs”. A motif is a short segment in the DNA sequence, between 6–20 nucleotide pairs, usually fewer than 10, that can be positioned at different locations within the regulatory region of a gene [7]. Tanay et al. [8] focus on identifying motifs that are “enriched”, i.e., the motif occurs in multiple species, controlling analogous regulons in those species.

Sarda and Hannenhalli [9] present a method for detecting rewiring, switching one transcription factor to another transcription factor in the same 23 yeast species we investigate.

Nocedal and Johnson [7] analyze more complex cases of transcription factor rewiring in yeast and concludes that future research is needed to understand transcription factor rewiring in regulatory networks that involve *multiple transcription factors and larger regulons*. They also say that it is important to consider evolution in the study of transcription factor rewiring. For us that means considering how regulation in a branch differs from the regulation in the other species of the tree. Our algorithm automatically finds a collection of genes for which switching occurs.

**What our method does** While it has been demonstrated that one transcription factor can be replaced by another (e.g., [2]), our algorithm looks for larger scale replacements. We present the first computational method that finds a regulon (denoted *G*) and two teams of transcription factors (denoted *T* and *T* ^∗^) for which there has been rewiring over evolutionary time for a specified branch *M* of the phylogenetic tree.

## Methods

### Data

We use 53 evolutionarily conserved co-expression modules detected in [10] based on *S. cerevisiae* and *C. albicans*. Additional file 1: Our supplementary material lists the genes in each module (those modules for which there was a full set of orthologs for all the species). Some modules are contained in larger modules. The number of genes in each *S. cerevisiae* module ranged from 1 to 614 with an average of 54 and a total of 2840 genes for all the *S. cerevisiae* modules. We study the 23 *Ascomycota* fungi species with an established phylogenetic tree from [8] shown in Fig. 1. Our yeast species includes *Saccharomyces cerevisiae*, *Candida albicans* and *Ashbya gossypii*. All 23 yeast species names are provided in Additional file 2: Supplementary material.

We used the orthology mapping of corresponding genes across the 23 yeast species from [11]. In some cases there is no gene for a given species, but we chose genes that had the representatives (or orthologs) in all or almost all of the species. “Orthologs” are genes in related species that have similar nucleotide sequences, suggesting they came from the same ancestral gene by speciation. When a gene has multiple copies in one species, we pick one copy at random, resulting in 2557 genes of *S. cerevisiae* – plus the orthologous genes across the other 22 *Ascomycota* species.

This paper is based on our calculation and analysis of transcription factor binding probabilities, the computed probability that a transcription factor binds somewhere in the 600-base region preceding a gene of one of our species (we obtained those regions from [11]). We refer to that region as the “upstream promoter region”. The set of 126 yeast transcription-factor-DNA binding-motifs (represented as Positional Weight Matrices (PWM)) was obtained from Transfac DB Database [12, 13]. While there are many factors determining whether a gene is activated or deactivated, it seems likely to be significant if the probability of a transcription factor is high for a branch of the phylogenetic tree and lower for the remaining species, or vice versa. We computed a binding probability for each of 126 transcription factors binding to each of 2557 genes in each of 23 *Ascomycota* species for a total of approximately 126×2557×23 probabilities, i.e., approximately 7 million probabilities (provided in Additional file 3: Supplementary material). Each of the genes that we selected was present in *S. cerevisiae*. We used the same 23 Ascomycota fungi and phylogeny [8], and our set of 126 transcription factors includes most of the 88 transcription factors that [14] uses, so we safely use 126 transcription factor binding motifs associated to S. cerevisiae and applied them to the other yeast species as [14] has demonstrated that most transcription factors have conserved their DNA motifs over large evolutionary distances.

### Our skewness method

For each species, gene, and transcription factor, we examine the “*binding probability*”, the probability that the transcription factor binds to the upstream promoter region of the gene.

If a particular branch of the phylogenetic tree has been selected, we say a transcription factor-gene pair is *(positively) skewed toward that branch* if the binding probabilities are on the average higher for species in that branch than for the species in the complement. Later we will define our function *skew* that measures how much it is skewed; (see Eq. 3). We say the pair is *negatively skewed toward a branch* if the reverse is true, that the binding probabilities are lower for the branch than in the complement. We usually average the skewness of a transcription factor over a collection of genes.

**Computing skewness** We pick a group *M* of species representing some branch of species in the phylogenetic tree in Fig. 1 (e.g., species 1−10). We use *M* ^⋆^ to designate the remaining species, 11−23 in this case. Hence *M* defines a branch (or partition) of species in the tree.

All calculations use some choice of *M* but we often omit mention of *M* and *M* ^⋆^ to simplify the notation.

For a collection of genes *G* we say a transcription factor is skewed towards *M* if it binds more strongly (averaging over the genes in *G*) for species in *M* than for species in *M* ^⋆^, and similarly it is skewed towards *M* ^⋆^ if the reverse holds. We aim at finding a branch and some related genes *G* in some module *R* and two collection of transcription factors that we denote *T* and *T* ^⋆^ so that on the average, transcription factors in *T* are skewed towards *M* for genes in *G*, while transcription factors in *T* ^⋆^ are skewed towards species in *M* ^⋆^.

To make that precise, we define the skewness, a measure of the difference in the average binding probabilities between *M* and *M* ^⋆^. Specifically, for a given branch *M* (with complement *M* ^⋆^) and each transcription factor *x* and each gene *g*, we compute the **skewness** *skew*(*x*,*g*,*M*) as follows in Eq. 4. We write 〈… 〉 for an average. We note that the average binding probability is computed by averaging over those species that have an ortholog of *g*; we exclude those species that do not have an orthologous gene from the average. All of the following depend on the choice of *M*. First we define *P*_*x*,*g*,*s*_= the binding (or occupancy) probability for transcription factor *x* to bind to the promoter of gene *g* in species *s*. (See Additional file 4: Supplementary methods, Section: Estimating transcription factor binding probabilities). We will use “ ^∗^” to indicate *M* ^∗^, the complement of *M* is being used in a calculation.

Now we present a formula for the extent to which the binding probability of one transcription factor to one gene is “skewed”, that is, stronger on the species in *M* than in *M* ^⋆^,

1 $$ skew(x,g,M) = \langle P_{x,g,s}\rangle_{s\in M} - \langle P_{x,g,s}\rangle_{s\in M^{\star}},  $$

Here “skew” measures how much *x* is skewed towards *M* for *g*. It is greater than 0 if *x* is skewed towards *M* and is less than 0 if *x* is skewed towards *M* ^⋆^.

**Fig. 2:**
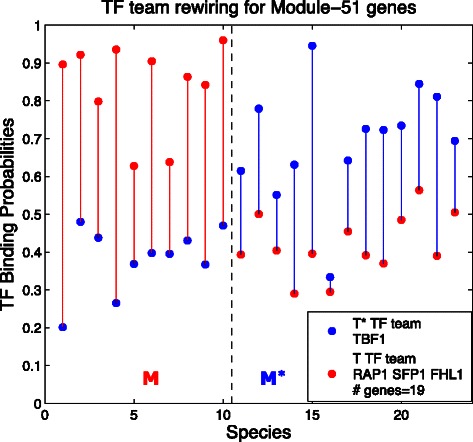
Transcription factor rewiring for Module 51, Ribosomal Protein (RP) genes. Here we describe the meaning of this and several following graphs. The species tree is partitioned into two groups: *M* is the set of species in one branch (labeled “10” in Fig. 1) and *M* ^⋆^ consists of the rest of the 23 species. In this and related figures, a dashed vertical line (or two) separates *M* from *M* ^⋆^. For each of the 23 species on the horizontal axis, we plot two dots, each of which is an average of binding probabilities that a transcription factor binds to a gene. Here for example each red dot is the average of 57 (=3 transcription factors in the red team times 19 genes) binding probabilities for the species in question, i.e., averaging over the genes in *G* and the transcription factors in team *T* (*red dots*) or in team *T* ^⋆^ (*blue dots*). The two dots for each species are connected with a solid line using the color of the upper dot. The first row in Table 1 reports on this case. Note that the box in the *lower right* specifies first the *blue* team *T* ^⋆^ (which here consists of a single transcription factor, TBF1), then the *red* team *T* (which here consists of three transcription factors, namely RAP1 SFP1 and SFL1), and finally the number of genes in the block. When there are too many transcription factors to fit in the box, only a few are given, but full data is given in the Additional file 5: Supplementary material for this graph (and all related graphs) including the names of the 19 genes that are discussed here

Figure 2 is a prime example of our findings. It shows what we find when we investigate the module of RP genes focusing on the branch of the phylogeny tree denoted by ‘10’ in Fig. 1 and consisting of the leftmost 10 species in that Figure. The dashed vertical line separates that branch from the rest of the tree. We see that for the 19 genes, transcription factor TBF1 (blue dots) has generally lower binding probabilities in *M* than in *M* ^⋆^ while the three transcription factors (the red team) are higher in *M* than in *M* ^⋆^ for those genes. Hence the overall dominance between the two teams is opposite for the red and blue teams. Note that the literature discusses this kind of switch for transcription factor TBF1 versus transcription factor RAP1 (a member of the red team), but here we find the switch apparently involves two other transcription factors as well, transcription factor FHL1 and transcription factor SFP1, members of the red team.

**Table 1:** Finding max blocks

Module	Part. *M*	Skew	Dif(*M* ^⋆^)	Dif(*M*)	# *T* ^⋆^:#*T*	#G/#MG	Figure
51	10	0.734	–0.276	0.457	1:3	19/31	2
59	112	0.727	–0.241	0.486	9:1	2/2	3
55	4	0.726	–0.112	0.615	14:12	1/1	4
40	20	0.699	–0.712	–0.013	2:33	3/12	5
56	113	0.697	–0.165	0.531	4:12	1/1	6
42	113	0.655	–0.147	0.507	6:9	1/1	–
52	4	0.642	–0.037	0.604	6:2	7/24	–
16	112	0.630	–0.020	0.650	18:1	16/24	–
31	112	0.600	0.098	0.699	19:2	2/2	–
35	4	0.598	–0.012	0.586	10:17	1/1	–
53	112	0.583	0.003	0.612	21:1	20/24	–
62	20	0.580	–0.426	0.154	5:3	27/74	–

The first row (Module 51 and branch 10) describes the max block found for this module and branch. The column “Skew” is the max block’s skewness score *skew*(*T*,*T* ^⋆^,*G*)=0.734; next is the difference between the averages of *T* and *T* ^∗^ on the species in *M* ^∗^, i.e., Dif(*M* ^⋆^))=0.457, followed by the corresponding difference for the species in *M* is Dif(*M*)=−0.276. The column # *T* ^⋆^:#*T* reports the numbers of transcription factors in *T* ^∗^ and *T*; and the column #G/#MG reports the number of genes in the regulon compared with the number in the module. The column “Figure” lists the figure number corresponding to the module or ’-’ for modules without figures. The cases shown have the highest *skew* scores and are listed in order of those scores. When a module has similar results for branches that only differ slightly, we show only the one whose block has the highest skewness. A more extensive set of data is included in Additional file 5: Supplementary material

We also define the skewness for a collection *T* of transcription factors, a collection of genes *G*, and a branch *M* as follows by averaging the *skew*(*x*,*g*) all the the transcription factors *x* in *T* and all the genes *g* in *G*, as follows.

2 $$ skew(T,G) = \langle skew(x,g)\rangle_{x\in T,g\in G}.  $$

For each branch *M* and Module *R* our goal is to identify a group *G* of genes in *R* and two teams or groups of transcription factors *T* and *T* ^⋆^ so that

3 $$ skew(T,T^{\star},G)= skew(T,G)- skew(T^{\star},G)  $$

is large. In the cases we care about, *skew*(*T*,*G*)>0 and *skew*(*T* ^⋆^,*G*)<0.

### Algorithms

#### Terminology

##### Blocks and substitution-maximizing blocks

We define a block denoted (*T*,*T* ^∗^,*G*) to be two groups or teams *T* and *T* ^∗^ of transcription factors and a group G of genes. We say there is a *rewiring* for a branch *M* of the tree when transcription factors in T are positively skewed for species in *M* for the genes in *G* while the transcription factors in *T* ^∗^ are negatively skewed.

We define a *“substitution-maximizing block”* or more simply a *max block* to be a block which has the property that if we substitute any gene for one of the genes in *G*, or any transcription factor for one of the transcription factors in the teams, then the skewness cannot not increase. But discarding a low scoring gene or transcription factor would raise the score of the block. Indeed the blocks with the highest scores are those that that have exactly one gene and one transcription factor in each of *T* and *T* ^∗^.

Finding max blocks by enumerating subsets is clearly out of the question, since we are dealing with candidate sets that may have dozens of genes and dozens of transcription factors.

We can refer to a block (*T*,*T* ^∗^,*G*) as an (*m*,*m* ^∗^,*m*_*G*_)-block when *m*,*m* ^∗^, and *m*_*G*_ are the numbers of elements in *T*,*T* ^∗^, and *G* respectively.

##### Overview

For any starting collection *G*_0_ of *m*_*G*_ genes, the ping-pong algorithm finds some sets *T* and *T* ^∗^ and eventually a max block (*T*,*T* ^∗^,*G*) by repeatedly making substitutions in the elements of *T*,*T* ^∗^, and *G* that increase the score *skew*(*T*,*T* ^∗^,*G*); and since only substitutions are made, the numbers of elements in *T*,*T* ^∗^, and *G* remain *m*,*m* ^∗^, and *m*_*G*_ respectively. A gene or transcription factor that is eliminated from one of the sets at one stage may later return after the mix of genes and transcription factors has changed.

##### A sequence of ever-shrinking max blocks

Next one of the numbers *m*,*m* ^∗^, and *m*_*G*_ is decreased by 1: the discussion of “importance” below describes which of these is decreased. This decrementing process continues, yielding a sequence of max blocks whose total *m*+*m* ^∗^+*m*_*G*_ decreases in steps of 1. When the process is stopped depends on the needs of the user. As discussed below, here we chose to stop when the importance (a ratio) reaches 0.5.

##### Our Ping-Pong Algorithm that yields a max block

In the game of ping-pong, the ball goes back and forth between the two sides. Here the block goes back and forth between two steps. The ping-pong algorithm consists of alternating between steps *TF* and *G* below repeatedly with *skew*(*T*,*T* ^∗^,*G*) increasing at each step until the process stops in the sense that skew reaches an equilibium, a max block.

A key point is that *T* and *T*∗ are generated from *G* without knowledge of previous versions of *T* and *T*∗. Similarly *G* is generated purely from *T* and *T*∗ without reference to any previous versions of *G*.

The ping-pong algorithm requires three positive integers, *m*,*m* ^∗^,*m*_*G*_ and a set *G* of *m*_*G*_ genes in a regulon R. The first time the ping-pong algorithm is applied, *m*_*G*_ is the number of genes in the Module *R* and *m*+*m* ^∗^ is the total number of transcription factors. At least one of these three numbers will decrease during the attrition step described below.

##### Step TF: choosing transcription factors *T* and *T*^∗^

Given a set *G* of genes, we compute the *skew*(*x*,*G*) scores of every transcription factor x and let the new *T* be the *m* highest scoring transcription factors and let *T* ^∗^ be the *m* ^∗^ lowest scoring transcription factors. Since *skew*(*T*,*G*) is the average of *skew*(*x*,*G*) for *x* in *T*, it follows that *skew*(*T*,*G*) is increased (or equal) by this new *T*. Similarly −*skew*(*T* ^∗^,*G*) is increased by the new choice of *T* ^∗^ and so is *skew*(*T*,*T* ^∗^,*G*).

##### Step G. choosing G

Note that *skew*(*T*,*T* ^∗^,*G*) is the average over the *m*_*G*_ genes in *G* of the terms

$$skew(T,g)-skew(T^{*},g). $$

Next compute that term for each gene *g* in *R* and we set the new *G* to be the *m*_*G*_ highest scoring genes in *R*. That increases (or possibly makes no change) in *skew*(*T*,*T* ^∗^,*G*).

Lemma: Steps G and TF never decrease the skew score.

To see this, let *m* be the number of transcription factors in *T* and *m*_*G*_ be the number of genes in *G*. Notice that *skew*(*T*,*G*) can be written three ways, namely as the average of the *m* terms *skew*(*x*,*G*), averaging over all *x* in *T*, or as the average of the *m*_*G*_ terms *skew*(*T*,*g*), averaging over all *g* in *G*. Both are equal to the average of the *m*×*m*_*G*_ items *skew*(*x*,*g*).

4 $$ skew(T,G) = \langle skew(X,g)\rangle_{g\in G} = \langle skew(x,G)\rangle_{X\in T}  $$

Hence if any gene *g* is introduced by Step *G*, it must have a higher skew scores

$$skew(T,T^{\star},g) = \langle skew(T,g)\rangle_{g\in G} - \langle skew(T^{\star},g)\rangle_{g\in G} $$

than each gene that is replaced. Similarly each transcription factor changed by step TF must increase the skew score.

In the above transcription factor step the algorithm is supposed to select the highest *m* scoring transcription factors for *T*, but for some choices of *G* there are fewer than *m* that have positive scores, or similarly with *T* ^∗^ there can be too few with negative scores. In such cases we terminate the ping-pong run. There are ways around this as long as there are some transcription factors with positive scores and others with negative scores: just decrease *m* or *m* ^∗^ as needed, but our goal was to present the algorithm in its simplest form. It is also possible to encounter sets of genes *G* for which there are no transcription factors with positive scores or none with negative scores.

##### Ping-pong stops at a max block

After applying this algorithm repeatedly, there will be no substitution of a single transcription factor or a single gene that would increase *skew*(*T*,*T* ^∗^,*G*) so that *T*,*T* ^∗^,*G* is a max block.

The algorithm alternates back and forth between the two steps repeatedly, letting *T* and *T* ^∗^ determine the set of genes *G*, and then letting *G* determine transcription factor teams *T* and *T* ^∗^. Each step increases the overall score *skew*(*T*,*T* ^∗^,*G*) until it stops at a max block: the only changes in the sets are those that increase the overall score. Since there are only a finite number of choices, the procedure must eventually stop at a max block, where the *G* that is used in step G is the *G* that is produced in the TF step.

**Ping-Pong pseudocode**

**Input:** (*all*_*Gs*,*G*_0_,*all*_*TFs*,*m*,*m* ^⋆^,*mG*): *all*_*Gs* is the set of all genes in some module *R*; *G*_0_ is an initial gene set of mG genes; *all*_*TFs* is the set of all transcription factors; *m*,*m* ^⋆^,*mG* remain constant; and *m*,*m* ^⋆^ are the numbers of transcription factors in team *T* and *T* ^⋆^ and *mG* is the number of genes

**Output**: The output is the max block (*G*, *T*, *T* ^⋆^) and its skewness score

1. *new*_*score*=0
2. *G*=*G*_0_
3. **Do**
4. *score*=*new*_*score*
5. Compute *Step TF*: Choose transcription factor teams *T* and *T* ^∗^
6. Compute *Step G. Choose genes G*
7. *new*_*score*=*skew*(*T*,*T* ^⋆^,*G*)
8. **while** *new*_*score*>*score*: (score is increasing)
9. return G, T, *T* ^⋆^, score
10. **Stopping condition:** Neither Step G nor Step TF ever decreases the skew score, so it must reach an equillibrium

##### The attrition step

For each *x* in *T* we define the “importance” of *x* to be the ratio of *skew*(*x*,*G*) divided by the highest score of the transcription factors in *T*; similarly for each *y* in *T* ^∗^, the “importance” of *y* is the ratio of *skew*(*y*,*G*) divided by the lowest score of the transcription factors; and for each *g* in *G*, the “importance” of *g* is the ratio of *skew*(*T*,*T* ^∗^,*g*) divided by the highest score in *G*. We now compare all of the importance scores and delete the one with the lowest score. In other words, we decrease by 1 one of the *m*,*m* ^∗^,*m*_*G*_. That increases the overall *skew* score. Now again we play ping-pong with the new reduced numbers, starting the game with our current *G*, possibly reduced by one gene.

As we proceed decreasing the numbers, we may lose some transcription factor or gene that later becomes more important to a reduced set of genes and transcription factors and so it enters back in. That is why we choose new teams from all transcription factors, not just the ones that were included on the last step, and the same holds for genes, using any genes in the specified regulon. We compute binding probabilities with 8 digit precision to avoid having tie scores, but if there is a tie score and one transcription factor or gene must be chosen, we retain the one(s) that comes first alphabetically.

##### When should attrition stop?

When we start, it is likely that some skew scores will be near 0, much smaller than other skew scores, so their importance will be near 0. The scientist who wishes to find many involved interacting genes and transcription factors might stop when the importance has risen to 0.25 (meaning that all the importance scores lie between 0.25 and 1.0). The experimentalist might wish to deal with fewer transcription factors and genes and so might stop at 0.75. In this paper and in the Additional file 5: Supplementary material we stopped when the importance reached 0.5.

## Results

We have examined the 12 largest branches of the species tree for each of the above mentioned modules using this approach. We indicated the branches with a slash and labeled them with a number as shown on Fig. 1. We determined a “max block” for each module and branch. For some, we found strong indications of rewiring.

Table 1 shows the cases with the largest skewness for the block of the module and branch, sorted by descending skewness. Columns 4 shows the difference Dif(*M* ^⋆^) between the two teams, *T* and *T* ^⋆^, on *M* ^⋆^,

5 $$ \begin{aligned} \text{Dif}(M^{\star})&=\text{Dif}^{\star}(T,T^{\star},G) \\ &=\langle P_{x,g,s}\rangle_{x\in T,g\in G,s\in M^{\star}} - \langle P_{x,g,s}\rangle_{x\in T^{\star},g\in G,s\in M^{\star}} \end{aligned}  $$

while column 5 shows the difference Dif(*M*) on *M*,

6 $$ \begin{aligned} {}\text{Dif}(M)&=\text{Dif}(T,T^{\star},G) \\&= \langle P_{x,g,s}\rangle_{x\in T,g\in G,s\in M} - \langle P_{x,g,s}\rangle_{x\in T^{\star},g\in G,s\in M} \end{aligned}  $$

**Transcription factor rewiring for Module-51 genes** Module 51 (see Fig. 2) consists of Ribosomal Protein (RP) genes exclusively. In the Introduction we noted that [2] reported that one transcription factor substitutes for another on some collection of genes in two species, namely Rap1 in *Saccharomyces cerevisiae* substitutes for TBF1 in *Candida albicans* for ribosomal RP genes. We find for a branch of 10 species, RAP1, FHL1, and SFP1 substitute for TBF1. Indeed we find that their skewness scores are similar: *skew*(*Tbf*1,*Rap*1,*G*)=0.777;*skew*(*Tbf*1,*Fhl*1,*G*)=0.713;*skew*(*Tbf*1,*Sfp*1,*G*)=0.711, where our algorithm finds the regulon *G* consists of 19 of the 31 RP genes in Module 51. See the Additional file 5: Supplementary material for a list of the 19 genes and other detailed information about the most significant block that was found for each module. Note that FHL1 is mentioned in [6] as a “a key player” in the regulation of RP genes in S. cerevisiae. We find it is involved in rewiring, according to our calculations.

**Fig. 3:**
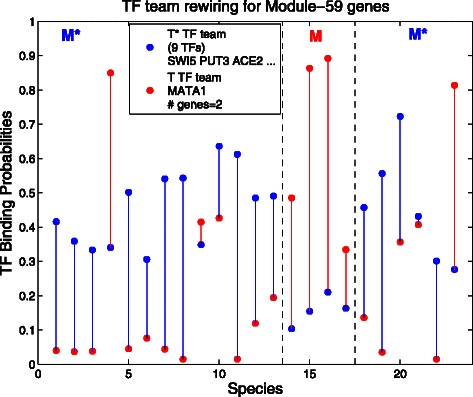
Transcription factor rewiring for Module-59 (RNA methylation) genes. Here *M* is branch 112 from the phylogeny tree, so *M*={14,⋯,17} and *M* ^⋆^={1,⋯,13;18,⋯,23}. In *Mred* dominates blue, while elsewhere *blue* mostly dominates red

**Transcription factor rewiring for Module-59 genes** Module 59 consists of conserved, co-expressed genes related to the biological function *RNA methylation*. Here there are two genes in the module and both are in the rewiring block. In Fig. 3 we see a much more complicated apparent rewiring than in Fig. 2.

What is striking is that in 13 of the 19 species in *M* ^⋆^ transcription factor MATA1 (red dots) has binding probabilities near 0, (though in two *M* ^⋆^ species it is high). In contrast in the branch *M*, it is higher than the *T* ^⋆^ team (blue dots, consisting of 9 transcription factors.

**Transcription factor rewiring for the Module-55 gene YMR290C** Module 55 consists of a conserved, co-expressed gene related to the biological function *ribosomal subunit assembly*.

**Fig. 4:**
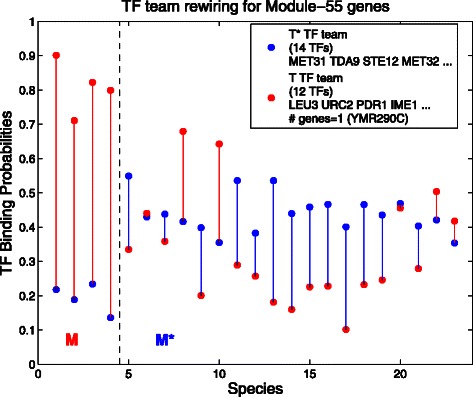
Transcription factor rewiring for Module-55 genes. Here *M* is branch 4 from the phylogeny tree, so *M*={1,2,3,4}, and *M* ^⋆^={5,⋯,23}. Notice the large difference between *red* and *blue* dots in all species in *M*, while blue mostly dominates in *M* ^⋆^

Here in Fig. 4 if the tree is cut at the bottom, separating the right branch of 19 species from the left-most branch of 4, it is arbitrary as to which of the two branches is called *M* and we have called it the left branch. If however we had called it the right branch, the graph and results would be the same. What we see is that in the four species of *M*, the 12 transcription factors of the *T* team (red dots) very clearly dominate the 14 transcription factors of the *T* ^⋆^ team. In contrast on the right side, the binding probabilities of the two teams are much closer, apparently all active. So the apparent switching behavior here is that the *T* clearly dominates *T* ^⋆^ on the left, while on the right all the transcription factors interact at similar levels (remembering that each dot is only an average).

**Fig. 5:**
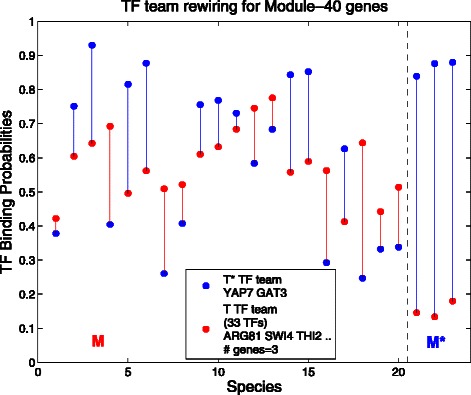
Transcription factor rewiring for Module-40 genes. Here *M* is branch 20 from the phylogeny tree, so *M*={1,⋯,20}, and *M* ^⋆^={21,⋯,23}. Branch 20 is special in that it cuts the tree at the root separating the tree into two branches: *M* ^⋆^ is also a branch. Hence the roles of *M* and *M* ^⋆^ can be switched and the *red* and *blue* colors could be reversed. Notice then that branch *M* ^⋆^ has a wide separation between red and blue, while for the rest of the species of the tree, red and blue are closer together

**Transcription factor rewiring for the Module-40 gene** Module 40 consists of conserved, co-expressed genes related to the biological function *actin cortical patch assembly*. The phenomenon seen in Fig. 5 is somewhat similar to what is seen in the previous figure for Module 55. The branch of three species has one team turned on and one turned off, or at least at much lower binding probabilities, while for each of the other species, the two teams have similar binding probabilities.

Here in Fig. 5 if the tree is cut at the top, separating the left branch of 20 species from the right-most branch of 3, it is arbitrary as to which of the two branches is called *M* and we have called it the left branch. What we see is that in the three species of *M* ^⋆^, the two transcription factors of the *T* ^⋆^ team (blue dots) very clearly dominates the 33 transcription factors of the *T* team for the 3 genes in the block. In contrast on the left side, the binding probabilities of the two teams are much closer, apparently all active. So the apparent switching behavior here is that the *M* ^⋆^ clearly dominates *M* on the right, while on the left all the transcription factors interact at similar levels (remembering that each dot is only an average).

**Fig. 6:**
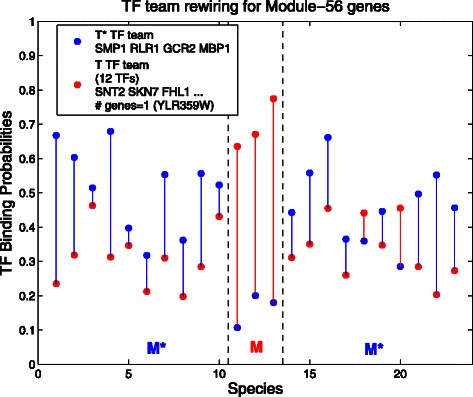
Transcription factor rewiring for Module-56 genes. Here *M* is branch 113 from the phylogeny tree, so *M*={11,⋯,13}, and *M* ^⋆^={1,⋯,10;14,⋯,23}. Notice the large difference between *red* and *blue dots* in all species in *M*, while blue mostly dominates in *M* ^⋆^

**Transcription factor rewiring for Module-56 genes** Module 56 (Fig. 6) consists of conserved, co-expressed genes related to the biological function *purine ribonucleotide biosynthetic process*. This example is most similar to Module 55 above in that there is an extreme difference between red and blue in *M* but not in *M* ^∗^.

## Discussion

Our method can address questions such as the following: Can different groups of genes in related species be regulated by the same group or “*team*” of transcription factors (as in Scenario 1)? Another question: Can a team of transcription factors become dominant for a collection of related genes in a tree branch while a second team is dominant on the other species (as in Scenario 2)? In this paper we focus in Scenario 2.

Our approach differs from that of Sarda and Hannenhalli [9] in that we define our skewness for each transcription factor while they define a function that compares the skewness of two transcription factors. They require more computation than our approach since they must make a complex computation of rewiring scores for each pair of transcription factors. We use extensive computation instead to look for more complicated situations where there can be several transcription factors that switch with one or more transcription factors. That is we find collections or teams of transcription factors which are positively skewed, averaging over the genes in a regulon, and for those transcription factors which are negatively skewed. We vary the selection of genes in the regulon and the teams of positively skewed transcription factors and the teams of negatively skewed transcription factors.

One of our colleagues, Chris Dock, tested a module (#2) of 40 genes. He picked *G*_0_ to have 36 randomly selected genes, and repeated this process 100 times. The process always arrived at the same max block (using importance = 0.5). That suggests the process is robust, but does not guarantee a unique result.

A module consists of related genes and one can imagine simplistically that the module represents a process with just two stages; first one set of genes is activated, and later another set. If there is rewiring, each might have its own max block, the union of which might be the max block that the above process finds. These can be found by using a modified approach, where instead of starting with a large set of genes and contracting it as we have described above, one can start with one gene and expand the collection of genes until importance 0.5 is reached. This ‘expanding“ approach would often yield a subset of the “contracting” approach and the subset would depend on the initial gene. Here we chose to keep our report simple by restricting attention to the expanding max block approach which gives an overview.

Note that while T* consists of the transcription factors for which skew(x,G) is smallest (most negative), they are not necessarily all negative, and we have excluded some cases where not all x in T* had negative skew score. This was an optional choice, but it seemed appropriate in view of the concept of rewiring.

## Conclusion

Nocedal and Johnson [7] write “We do not yet understand how a large network, composed of many transcription regulators and hundreds or thousands of target genes, forms in the first place.” We believe that considering only cases in which one transcription factor is switched with another will be inadequate to describe the evolution of networks. They also write “A change even in the regulation of a single gene can have important consequences in modern species’.... However, most biological processes require the coordinated expression of many genes rather than a single gene” to produce a useful phenotype.

Our investigation aims at providing a new approach to thinking about the very complex idea of rewiring, freeing us from the constraint of considering only one transcription factor substituting for one transcription factor (or one gene for one gene).

All the examples in this paper discuss rewiring (Scenario 2) via one team of transcription factors substituting for another team on a collection of genes. However, it is an equivalent problem mathematically - using the same set of binding probabilities - to have one team of genes substitute for another team of genes for a collection of transcription factors (the turnover problem, Scenario 1). In an example of a Scenario 1, Habib et al. [14] present a method for tracing the evolutionary history of regulatory interactions of 88 regulatory DNA motifs associated with transcription factors across 23 Ascomycota fungi, (the same 23 that we study). They use their method to explain the evolution of transcription factor turnover for a collection of genes. Here the transcription factor changes which genes it regulates while preserving the function of the genes. Gasch et al. [15] also study changes in which regulon members that are regulated by certain transcription factors.

We further expect to be able to investigate more complicated problems with very similar ideas in which there is simultaneously a rewiring of transcription factors and a turnover of genes.

No numerical investigation such as ours can produce definitive biological results, but the fact that our first case in Table 1, top row, is similar to a well known case is promising since our results find a team of three transcription factors instead of one in the published results. Table 1 shows the 12 cases with the highest *skew* scores, and for the top 5 we have included figures. These seem to suggest rewiring of teams of transcription factors. Of course it is desirable to have some of these cases checked experimentally.

It may be significant that eight of the twelve cases in Table 1 involve only two branches, namely branches 4 and 112. While branch 4 includes *Saccharomyces cerevisiae* and relatives, branch 112 includes *Candida albicans* and relatives. Those species have been reported in cases of rewiring in the literature.

**Fig. 7:**
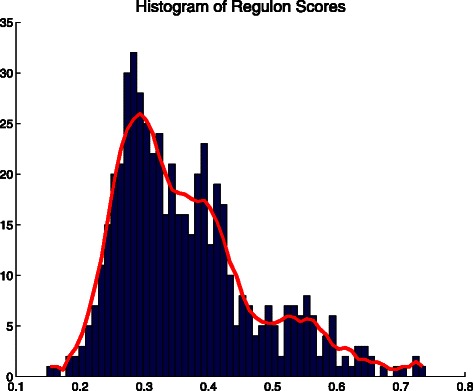
Histogram of Skewness Scores. The histogram of all skewness scores for the max blocks of all modules and all branches is shown in *blue*. The *horizontal axis* reports the two-digit truncation of *skew* and the height is the number of max blocks that had that score. The moving average in a sliding window of size 7 is shown in *red*. Note the long tail on the right, which corresponds to the high *skew* max blocks that we are most interested in

For each module and each branch of the tree we have computed a block (except for a small number of cases). The existence of a max block is not evidence of significant rewiring, and in fact there is no apparent test of statistical significance. Our solution to this complication is to examine the blocks that have the greatest skewness. For each module and each branch of the tree we compute the skewness of the resulting max block. Figure 7 is the histogram. The distribution has a long tail consisting of high *skew* scores. We believe that several cases in this flat tail represent actual cases of rewiring.

We believe that the understanding of the evolution of transcription networks will have to invoke teams of transcription factors and teams of genes in some essential form.

## Additional files

Additional file 1 Supplementary material: all genes present in each module. We report all modules and for each module, we list all genes in the module. Each gene entry includes identifier and name. (PDF 91 kb)

Additional file 2 Supplementary material: yeast species. We report the yeast species names and identifier. (PDF 16 kb)

Additional file 3 Supplementary material: database of the computed transcription factor binding probabilities. We report the database of the computed binding probabilities of 126 transcription factors to 2557 genes shared by 23 species. (TXT 415 kb)

Additional file 4 Supplementary methods. We describe the method for estimating transcription factor binding probabilities for each PWM (equivalently, transcription factor) on the gene promoter, for each gene in each species. (PDF 135 kb)

Additional file 5 Supplementary material: the transcription factors and genes, and their skewness scores of the rewiring blocks. For each module, results are reported for its top scoring branch. Each module lists the highest scoring block. (PDF 155 kb)

Additional file 6 Supplementary material overview. We give an overview of the supplementary material and methods provided in this paper. (PDF 74 kb)
