# Supplementary material for: Evolution of transcriptional networks in yeast: alternative teams of transcriptional factors for different species
Source: BMC Genomics. 2016 Nov 11;17(Suppl 10):826. doi: 10.1186/s12864-016-3102-7 (PMC5123246; doi:10.1186/s12864-016-3102-7)
Supplement: Supplementary file 1 — Supplementary material: all genes present in each module. We report all modules and for each module, we list all genes in the module. Each gene entry includes identifier and name. (PDF 91 kb) [file 12864_2016_3102_MOESM1_ESM.pdf]

Supplementary Material:
All genes present in each module

For each module, all genes are reported.
Each gene entry includes id and name.

Module 1

gene:7, YAL003W;8, YAL005C;9, YAL012W;10, YAL014C;11, YAL015C;13, YAL020C;25, YAL039C;26, YAL043C;27, YAL044C;30, YAL058W;40, YAR042W;47, YBL011W;70, YBL069W;85, YBL098W;92, YBR004C;118, YBR066C;119, YBR067C;130, YBR085W;145, YBR118W;160, YBR145W;178, YBR210W;187, YBR240C;190, YBR244W;214, YBR291C;216, YBR296C;221, YCL009C;222, YCL010C;225, YCL025C;226, YCL027W;233, YCL050C;235, YCL055W;238, YCL064C;264, YCR060W;265, YCR063W;270, YCR075C;277, YCR089W;292, YDL030W;296, YDL037C;297, YDL039C;304, YDL048C;309, YDL059C;312, YDL066W;313, YDL069C;319, YDL081C;323, YDL087C;333, YDL120W;334, YDL125C;336, YDL130W;343, YDL140C;344, YDL141W;350, YDL160C;360, YDL182W;368, YDL198C;380, YDL220C;384, YDL231C;416, YDR044W;417, YDR046C;418, YDR050C;420, YDR055W;426, YDR077W;447, YDR130C;465, YDR174W;472, YDR192C;488, YDR227W;495, YDR252W;496, YDR253C;504, YDR268W;542, YDR362C;552, YDR382W;554, YDR385W;562, YDR405W;575, YDR430C;576, YDR432W;583, YDR448W;599, YDR470C;611, YDR487C;618, YDR498C;623, YDR510W;631, YDR538W;635, YEL004W;636, YEL006W;640, YEL016C;641, YEL017W;643, YEL022W;652, YEL046C;653, YEL047C;663, YEL065W;664, YEL066W;666, YEL071W;673, YER011W;676, YER018C;687, YER042W;688, YER044C;691, YER052C;696, YER058W;698, YER062C;709, YER088C;710, YER089C;711, YER090W;712, YER091C;719, YER104W;722, YER109C;728, YER124C;735, YER132C;744, YER153C;760, YER180C;761, YER183C;767, YFL010C;775, YFL031W;796, YFR034C;802, YFR055W;806, YGL009C;818, YGL032C;822, YGL049C;825, YGL055W;826, YGL056C;830, YGL065C;837, YGL089C;838, YGL090W;853, YGL116W;858, YGL125W;859, YGL126W;869, YGL143C;877, YGL162W;888, YGL179C;899, YGL209W;907, YGL226W;922, YGL254W;923, YGL255W;934, YGR036C;935, YGR037C;936, YGR041W;942, YGR055W;946, YGR062C;951, YGR075C;953, YGR077C;964, YGR096W;976, YGR133W;978, YGR138C;987, YGR156W;998, YGR174C;1002, YGR180C;1006, YGR192C;1015, YGR212W;1030, YGR257C;1032, YGR261C;1041, YGR282C;1043, YGR286C;1050, YHL009C;1057, YHL025W;1059, YHL028W;1071, YHR011W;1075, YHR018C;1082, YHR036W;1088, YHR046C;1089, YHR047C;1091, YHR053C;1092, YHR058C;1110, YHR086W;1121, YHR116W;1138, YHR167W;1143, YHR179W;1144, YHR183W;1171, YIL022W;1178, YIL044C;1181, YIL049W;1189, YIL074C;1195, YIL093C;1201, YIL106W;1206, YIL114C;1208, YIL116W;1210, YIL119C;1212, YIL121W;1218, YIL140W;1220, YIL145C;1226,

YIL154C;1248, YIR033W;1249, YIR034C;1261, YJL026W;1269, YJL044C;1275, YJL056C;1285, YJL078C;1286, YJL079C;1292, YJL088W;1298, YJL102W;1348, YJR010W;1351, YJR017C;1372, YJR077C;1381, YJR109C;1392, YJR137C;1394, YJR147W;1395, YJR150C;1399, YKL001C;1422, YKL043W;1423, YKL051W;1435, YKL081W;1437, YKL084W;1439, YKL092C;1441, YKL096W;1465, YKL164C;1482, YKL204W;1520, YKR062W;1534, YKR093W;1544, YLL010C;1548, YLL024C;1550, YLL028W;1563, YLL052C;1596, YLR069C;1611, YLR109W;1619, YLR121C;1631, YLR150W;1656, YLR214W;1662, YLR228C;1669, YLR242C;1671, YLR245C;1672, YLR249W;1678, YLR265C;1687, YLR286C;1703, YLR340W;1706, YLR348C;1721, YLR390W;1748, YLR452C;1775, YML051W;1814, YMR011W;1829, YMR042W;1836, YMR058W;1843, YMR073C;1859, YMR112C;1860, YMR113W;1884, YMR182C;1885, YMR183C;1886, YMR186W;1904, YMR222C;1926, YMR264W;1933, YMR276W;1945, YMR303C;1950, YMR316W;1952, YMR318C;1953, YMR319C;1962, YNL014W;1975, YNL052W;1981, YNL065W;1986, YNL072W;1990, YNL078W;1992, YNL080C;2002, YNL104C;2019, YNL145W;2021, YNL148C;2022, YNL152W;2027, YNL169C;2030, YNL180C;2066, YNL259C;2076, YNL277W;2092, YNL309W;2118, YNR033W;2121, YNR037C;2123, YNR041C;2125, YNR044W;2134, YNR057C;2137, YNR067C;2141, YOL002C;2151, YOL026C;2153, YOL039W;2156, YOL042W;2160, YOL058W;2161, YOL059W;2182, YOL101C;2187, YOL110W;2208, YOL155C;2238, YOR071C;2242, YOR077W;2245, YOR083W;2254, YOR107W;2256, YOR109W;2263, YOR133W;2280, YOR187W;2284, YOR202W;2293, YOR226C;2295, YOR231W;2332, YOR334W;2334, YOR337W;2337, YOR344C;2341, YOR360C;2349, YOR382W;2350, YOR383C;2358, YPL015C;2366, YPL036W;2373, YPL048W;2374, YPL049C;2376, YPL058C;2378, YPL061W;2397, YPL111W;2420, YPL156C;2454, YPL221W;2469, YPL265W;2474, YPL274W;2476, YPR002W;2493, YPR035W;2510, YPR080W;2518, YPR106W;2524, YPR122W;2525, YPR124W;2536, YPR156C;2550, YPR183W;2554, YPR192W;2556, YPR194C

Module 2

gene:40, YAR042W;89, YBL106C;135, YBR102C;287, YDL019C;444, YDR122W;445, YDR126W;459, YDR164C;461, YDR166C;543, YDR363W-A;671, YER008C;681, YER031C;766, YFL005W;777, YFL039C;880, YGL167C;900, YGL210W;911, YGL233W;1067, YHR001W;1105, YHR073W;1187, YIL068C;1209, YIL118W;1217, YIL138C;1290, YJL085W;1336, YJL204C;1433, YKL079W;1451, YKL129C;1492, YKR003W;1507, YKR031C;1603, YLR096W;1633, YLR166C;1663, YLR229C;1857, YMR109W;1991, YNL079C;2057, YNL243W;2074, YNL272C;2085, YNL293W;2297, YOR237W;2413, YPL145C;2466, YPL249C;2490, YPR032W;2500, YPR055W;2514, YPR095C

Module 5

gene:5, Q0140;7, YAL003W;8, YAL005C;12, YAL016W;22, YAL035W;48, YBL013W;55, YBL027W;57, YBL038W;64, YBL057C;72, YBL072C;75, YBL076C;78, YBL080C;80, YBL087C;84, YBL092W;102, YBR031W;109, YBR048W;116, YBR061C;125, YBR079C;128, YBR084C-A;134, YBR101C;145, YBR118W;147, YBR120C;148, YBR121C;159, YBR143C;161, YBR146W;171, YBR181C;173, YBR189W;174, YBR191W;195, YBR251W;200, YBR261C;218, YCL001W-A;219, YCL001W-B;229, YCL037C;241, YCR003W;248, YCR024C;251, YCR031C;267, YCR071C;294, YDL033C;301, YDL044C;311, YDL061C;313, YDL069C;316, YDL075W;319, YDL081C;320, YDL082W;321, YDL083C;336, YDL130W;338, YDL133C-A;339, YDL134C;340, YDL136W;361, YDL184C;363, YDL188C;364, YDL191W;379, YDL219W;383, YDL229W;398, YDR012W;402, YDR023W;403, YDR025W;410, YDR037W;414, YDR041W;423, YDR064W;430, YDR091C;439, YDR115W;440, YDR116C;463, YDR172W;475, YDR197W;480, YDR211W;493, YDR237W;504, YDR268W;508, YDR283C;530, YDR337W;532, YDR341C;552, YDR382W;554, YDR385W;562, YDR405W;567, YDR418W;574, YDR429C;582, YDR447C;585, YDR450W;600, YDR471W;614, YDR494W;619, YDR500C;649, YEL034W;654, YEL050C;657, YEL054C;670, YER007C-A;679, YER025W;690, YER050C;695, YER056C-A;704, YER074W;708, YER087W;717, YER102W;725, YER117W;734, YER131W;744, YER153C;772, YFL022C;776, YFL034C-A;794, YFR031C-A;795, YFR032C-A;800, YFR049W;816, YGL030W;817, YGL031C;822, YGL049C;831, YGL068W;834, YGL076C;847, YGL103W;856, YGL123W;864, YGL135W;869, YGL143C;870, YGL147C;891, YGL189C;892, YGL190C;913, YGL236C;917, YGL245W;931, YGR027C;933, YGR034W;941, YGR054W;955, YGR083C;957, YGR085C;962, YGR094W;970, YGR118W;983, YGR148C;991, YGR162W;996, YGR171C;1003, YGR185C;1016, YGR214W;1018, YGR220C;1034, YGR264C;1046, YHL001W;1049, YHL004W;1054, YHL015W;1061, YHL033C;1070, YHR010W;1071, YHR011W;1076, YHR019C;1077, YHR020W;1078, YHR021C;1083, YHR038W;1096, YHR064C;1114, YHR091C;1129, YHR141C;1132, YHR147C;1139, YHR168W;1147, YHR189W;1153, YHR203C;1168, YIL018W;1183, YIL052C;1188, YIL069C;1191, YIL078W;1216, YIL133C;1223, YIL148W;1278, YJL063C;1294, YJL096W;1298, YJL102W;1313, YJL136C;1315, YJL138C;1326, YJL177W;1328, YJL189W;1329, YJL190C;1330, YJL191W;1346, YJR007W;1361, YJR047C;1375, YJR094W-A;1384, YJR113C;1386, YJR123W;1393, YJR145C;1401, YKL003C;1403, YKL006W;1424, YKL056C;1435, YKL081W;1456, YKL139W;1461, YKL155C;1462, YKL156W;1467, YKL167C;1468, YKL170W;1471, YKL180W;1479, YKL194C;1503, YKR026C;1516, YKR057W;1518, YKR059W;1529, YKR084C;1535, YKR094C;1546, YLL018C;1560, YLL045C;1570, YLR009W;1582, YLR029C;1586, YLR048W;1592, YLR060W;1593, YLR061W;1596, YLR069C;1597, YLR075W;1625, YLR139C;1634, YLR167W;1644, YLR185W;1648, YLR192C;1652, YLR203C;1672, YLR249W;1675, YLR262C-A;1677, YLR264W;1688, YLR287C-A;1691, YLR291C;1698, YLR325C;1701, YLR333C;1703, YLR340W;1704, YLR344W;1713, YLR367W;1717, YLR382C;1720, YLR388W;1727, YLR406C;1740, YLR439W;1741, YLR441C;1744, YLR448W;1756, YML009C;1762, YML024W;1763, YML025C;1764, YML026C;1766, YML028W;1780, YML063W;1783, YML073C;1790,

YML091C;1823, YMR023C;1840, YMR064W;1854, YMR097C;1863, YMR121C;1870, YMR142C;1871, YMR143W;1872, YMR146C;1875, YMR158W;1887, YMR188C;1890, YMR193W;1891, YMR194W;1908, YMR230W;1916, YMR242C;1921, YMR257C;1923, YMR260C;1939, YMR286W;1943, YMR293C;1947, YMR309C;1955, YNL001W;1956, YNL002C;1958, YNL005C;1962, YNL014W;1971, YNL040W;1983, YNL067W;1985, YNL069C;1987, YNL073W;1993, YNL081C;1998, YNL096C;2011, YNL122C;2024, YNL162W;2028, YNL177C;2029, YNL178W;2033, YNL185C;2040, YNL209W;2058, YNL244C;2060, YNL247W;2081, YNL284C;2088, YNL301C;2089, YNL302C;2090, YNL306W;2120, YNR036C;2121, YNR037C;2126, YNR045W;2150, YOL023W;2152, YOL033W;2153, YOL039W;2154, YOL040C;2180, YOL097C;2192, YOL120C;2193, YOL121C;2197, YOL127W;2200, YOL139C;2218, YOR014W;2234, YOR063W;2241, YOR076C;2248, YOR096W;2263, YOR133W;2267, YOR150W;2272, YOR167C;2273, YOR168W;2277, YOR182C;2280, YOR187W;2285, YOR204W;2290, YOR215C;2296, YOR234C;2306, YOR260W;2311, YOR276W;2316, YOR293W;2324, YOR312C;2333, YOR335C;2342, YOR361C;2345, YOR369C;2357, YPL013C;2369, YPL040C;2373, YPL048W;2382, YPL079W;2383, YPL081W;2389, YPL090C;2391, YPL097W;2395, YPL104W;2400, YPL119C;2407, YPL131W;2412, YPL143W;2423, YPL160W;2428, YPL173W;2435, YPL183W-A;2438, YPL190C;2441, YPL198W;2453, YPL220W;2463, YPL237W;2465, YPL249C-A;2481, YPR016C;2491, YPR033C;2495, YPR041W;2496, YPR043W;2497, YPR047W;2510, YPR080W;2511, YPR081C;2516, YPR102C;2527, YPR132W;2541, YPR163C;2543, YPR166C

Module 6

gene:1040, YGR280C;1151, YHR196W;1357, YJR041C;1912, YMR235C;1929, YMR269W;2286, YOR206W

Module 7

gene:18, YAL029C;153, YBR130C;382, YDL227C;433, YDR097C;1452, YKL130C;2073, YNL271C;2225, YOR035C;2281, YOR191W

Module 8

gene:19, YAL030W;135, YBR102C;335, YDL126C;459, YDR164C;461, YDR166C;526, YDR323C;597, YDR468C;618, YDR498C;671, YER008C;766, YFL005W;902, YGL212W;911, YGL233W;926, YGR009C;1060, YHL031C;1161, YIL004C;1187, YIL068C;1290, YJL085W;1480, YKL196C;1580, YLR026C;1598, YLR078C;1602, YLR093C;1633, YLR166C;1679, YLR268W;1885, YMR183C;1892, YMR197C;2147, YOL018C;2226, YOR036W;2240, YOR075W;2253, YOR106W;2329, YOR327C;2460, YPL232W;2500, YPR055W

Module 9

gene:52, YBL024W;116, YBR061C;329, YDL112W;369, YDL201W;442, YDR120C;460, YDR165W;823, YGL050W;1101, YHR070W;1308, YJL125C;1753, YML005W;1758, YML014W;1979, YNL062C;2127, YNR046W;2179, YOL093W;2195, YOL124C;2196, YOL125W;2202, YOL141W;2364, YPL030W

Module 10

gene:243, YCR010C;355, YDL170W;553, YDR384C;927, YGR019W;972, YGR121C;1241, YIR023W;2018, YNL142W;2102, YNR002C;2531, YPR138C

Module 11

gene:135, YBR102C;459, YDR164C;461, YDR166C;470, YDR189W;526, YDR323C;671, YER008C;842, YGL095C;911, YGL233W;1187, YIL068C;1290, YJL085W;1630, YLR148W;1633, YLR166C;1722, YLR396C;1909, YMR231W;2128, YNR049C;2500, YPR055W

Module 12

gene:202, YBR267W;268, YCR072C;392, YDR002W;430, YDR091C;457, YDR159W;785, YFR002W;840, YGL092W;844, YGL097W;845, YGL099W;1023, YGR245C;1109, YHR085W;1111, YHR088W;1152, YHR197W;1186, YIL063C;1207, YIL115C;1358, YJR042W;1476, YKL186C;1578, YLR022C;1692, YLR293C;1912, YMR235C;2249, YOR098C;2284, YOR202W;2286, YOR206W;2390, YPL093W;2481, YPR016C

Module 13

gene:150, YBR126C;391, YDR001C;406, YDR028C;434, YDR099W;602, YDR477W;625, YDR516C;637, YEL011W;693, YER054C;697, YER059W;736, YER133W;757, YER177W;789, YFR015C;801, YFR053C;852, YGL115W;863, YGL134W;1179, YIL045W;1182, YIL050W;1293, YJL089W;1317, YJL141C;1323, YJL164C;1478, YKL193C;1517, YKR058W;1674, YLR258W;1855, YMR105C;2275, YOR178C;2365, YPL031C;2452, YPL219W;2539, YPR160W

Module 15

gene:588, YDR454C;1769, YML035C

Module 16

gene:688, YER044C;803, YGL001C;807, YGL012W;944, YGR060W;1069, YHR007C;1104, YHR072W;1148, YHR190W;1324, YJL167W;1590, YLR056W;1605, YLR100W;1647, YLR189C;1746, YLR450W;1755, YML008C;1785, YML075C;1808, YML126C;1817, YMR015C;1894, YMR202W;1897, YMR208W;1903, YMR220W;2078, YNL280C;2124, YNR043W;2297, YOR237W;2398, YPL117C;2413, YPL145C

Module 17

gene:179, YBR212W;1482, YKL204W;1803, YML115C;2229, YOR043W

Module 18

gene:211, YBR284W;1281, YJL070C;1769, YML035C;2017, YNL141W

Module 19

gene:167, YBR166C;194, YBR249C;396, YDR007W;409, YDR035W;446, YDR127W;539, YDR354W;711, YER090W;813, YGL026C;871, YGL148W;1485, YKL211C;2095, YNL316C;2503, YPR060C

Module 20

gene:22, YAL035W;55, YBL027W;72, YBL072C;80, YBL087C;84, YBL092W;102, YBR031W;109, YBR048W;128, YBR084C-A;134, YBR101C;171, YBR181C;173, YBR189W;174, YBR191W;202, YBR267W;251, YCR031C;271, YCR077C;298, YDL040C;311, YDL061C;316, YDL075W;319, YDL081C;320, YDL082W;321, YDL083C;336, YDL130W;338, YDL133C-A;340, YDL136W;361, YDL184C;364, YDL191W;398, YDR012W;403, YDR025W;423, YDR064W;430, YDR091C;435, YDR101C;508, YDR283C;552, YDR382W;567, YDR418W;582, YDR447C;585, YDR450W;600, YDR471W;619, YDR500C;649, YEL034W;657, YEL054C;695, YER056C-A;704, YER074W;717, YER102W;725, YER117W;734, YER131W;776, YFL034C-A;787, YFR009W;794, YFR031C-A;795, YFR032C-A;816, YGL030W;817, YGL031C;834, YGL076C;847, YGL103W;856, YGL123W;864, YGL135W;870, YGL147C;891, YGL189C;895, YGL195W;931, YGR027C;933, YGR034W;941, YGR054W;957, YGR085C;970, YGR118W;983, YGR148C;1016, YGR214W;1046, YHL001W;1054, YHL015W;1061, YHL033C;1070, YHR010W;1073, YHR013C;1078, YHR021C;1129, YHR141C;1141, YHR170W;1153, YHR203C;1168, YIL018W;1183, YIL052C;1188, YIL069C;1216, YIL133C;1223, YIL148W;1237, YIR012W;1313, YJL136C;1326, YJL177W;1328, YJL189W;1329, YJL190C;1330, YJL191W;1361,

YJR047C;1375, YJR094W-A;1386, YJR123W;1393, YJR145C;1403, YKL006W;1436, YKL082C;1462, YKL156W;1471, YKL180W;1516, YKR057W;1535, YKR094C;1557, YLL039C;1560, YLL045C;1582, YLR029C;1586, YLR048W;1593, YLR061W;1597, YLR075W;1631, YLR150W;1634, YLR167W;1644, YLR185W;1670, YLR244C;1672, YLR249W;1677, YLR264W;1688, YLR287C-A;1698, YLR325C;1701, YLR333C;1703, YLR340W;1704, YLR344W;1713, YLR367W;1719, YLR387C;1720, YLR388W;1727, YLR406C;1741, YLR441C;1744, YLR448W;1762, YML024W;1764, YML026C;1780, YML063W;1783, YML073C;1861, YMR116C;1863, YMR121C;1870, YMR142C;1871, YMR143W;1891, YMR194W;1908, YMR230W;1916, YMR242C;1960, YNL007C;1962, YNL014W;1983, YNL067W;1985, YNL069C;1998, YNL096C;2024, YNL162W;2029, YNL178W;2049, YNL227C;2088, YNL301C;2089, YNL302C;2153, YOL039W;2154, YOL040C;2192, YOL120C;2193, YOL121C;2197, YOL127W;2234, YOR063W;2248, YOR096W;2272, YOR167C;2277, YOR182C;2296, YOR234C;2303, YOR253W;2316, YOR293W;2324, YOR312C;2345, YOR369C;2382, YPL079W;2383, YPL081W;2389, YPL090C;2407, YPL131W;2412, YPL143W;2441, YPL198W;2447, YPL211W;2453, YPL220W;2465, YPL249C-A;2495, YPR041W;2496, YPR043W;2516, YPR102C;2527, YPR132W

Module 21

gene:113, YBR055C;254, YCR035C;305, YDL051W;328, YDL111C;329, YDL112W;440, YDR116C;507, YDR280W;566, YDR416W;584, YDR449C;751, YER168C;864, YGL135W;916, YGL243W;963, YGR095C;989, YGR158C;1007, YGR195W;1090, YHR052W;1335, YJL203W;1515, YKR056W;1519, YKR060W;1595, YLR068W;1616, YLR117C;1638, YLR175W;1695, YLR312W-A;1774, YML046W;1790, YML091C;1824, YMR024W;1838, YMR061W;1883, YMR180C;1907, YMR229C;1914, YMR239C;2046, YNL221C;2084, YNL292W;2211, YOR001W;2219, YOR017W;2231, YOR048C;2283, YOR201C;2453, YPL220W

Module 22

gene:75, YBL076C;148, YBR121C;248, YCR024C;402, YDR023W;410, YDR037W;504, YDR268W;532, YDR341C;708, YER087W;772, YFL022C;849, YGL105W;917, YGL245W;962, YGR094W;996, YGR171C;1003, YGR185C;1034, YGR264C;1071, YHR011W;1076, YHR019C;1077, YHR020W;1114, YHR091C;1191, YIL078W;1479, YKL194C;1546, YLL018C;1717, YLR382C;1987, YNL073W;2060, YNL247W;2152, YOL033W;2180, YOL097C;2273, YOR168W;2369, YPL040C;2391, YPL097W;2395, YPL104W;2423, YPL160W;2491, YPR033C;2497, YPR047W;2511, YPR081C

Module 23

gene:258, YCR047C;373, YDL208W;731, YER127W;1632, YLR165C;1651, YLR197W;1906, YMR228W;2283, YOR201C;2470, YPL266W;2530, YPR137W

Module 24

gene:33, YAR002W;77, YBL079W;166, YBR165W;324, YDL088C;332, YDL116W;457, YDR159W;472, YDR192C;529, YDR335W;541, YDR361C;720, YER105C;721, YER107C;785, YFR002W;810, YGL016W;840, YGL092W;846, YGL100W;883, YGL172W;914, YGL238W;919, YGL247W;971, YGR119C;1017, YGR218W;1082, YHR036W;1186, YIL063C;1224, YIL149C;1268, YJL041W;1277, YJL061W;1358, YJR042W;1425, YKL057C;1430, YKL068W;1528, YKR082W;1702, YLR335W;1798, YML103C;1831, YMR047C;1866, YMR129W;1874, YMR153W;2035, YNL189W;2249, YOR098C

Module 25

gene:6, YAL002W;76, YBL078C;123, YBR077C;152, YBR128C;154, YBR131W;181, YBR217W;208, YBR280C;230, YCL038C;266, YCR068W;288, YDL020C;317, YDL077C;330, YDL113C;331, YDL115C;346, YDL149W;378, YDL216C;386, YDL234C;393, YDR003W;401, YDR022C;415, YDR043C;421, YDR059C;424, YDR069C;427, YDR080W;437, YDR108W;444, YDR122W;483, YDR219C;497, YDR255C;520, YDR313C;526, YDR323C;528, YDR330W;572, YDR425W;591, YDR460W;604, YDR479C;638, YEL012W;646, YEL030W;659, YEL060C;683, YER035W;716, YER101C;747, YER162C;790, YFR021W;804, YGL006W;857, YGL124C;875, YGL156W;879, YGL166W;889, YGL180W;908, YGL227W;937, YGR043C;939, YGR046W;992, YGR163W;1031, YGR258C;1047, YHL002W;1142, YHR171W;1150, YHR195W;1179, YIL045W;1197, YIL097W;1202, YIL107C;1203, YIL109C;1205, YIL112W;1267, YJL036W;1274, YJL053W;1289, YJL083W;1320, YJL155C;1323, YJL164C;1327, YJL178C;1356, YJR035W;1421, YKL041W;1494, YKR007W;1498, YKR019C;1512, YKR042W;1523, YKR076W;1559, YLL042C;1579, YLR025W;1600, YLR082C;1602, YLR093C;1606, YLR102C;1616, YLR117C;1630, YLR148W;1632, YLR165C;1640, YLR178C;1647, YLR189C;1668, YLR240W;1673, YLR254C;1680, YLR270W;1711, YLR360W;1732, YLR423C;1735, YLR431C;1813, YMR004W;1826, YMR030W;1844, YMR077C;1854, YMR097C;1858, YMR110C;1868, YMR135C;1869, YMR137C;1876, YMR159C;1880, YMR170C;1882, YMR174C;1892, YMR197C;1893, YMR201C;1909, YMR231W;2048, YNL223W;2056, YNL242W;2070, YNL265C;2103, YNR006W;2104, YNR007C;2175, YOL082W;2181, YOL100W;2186, YOL108C;2220, YOR023C;2225, YOR035C;2226, YOR036W;2228, YOR040W;2253, YOR106W;2262, YOR132W;2274, YOR173W;2300, YOR245C;2386, YPL084W;2387, YPL085W;2392, YPL100W;2401, YPL120W;2416, YPL149W;2422, YPL159C;2424, YPL166W;2498, YPR049C;2549, YPR181C;2552, YPR185W

Module 26

gene:50, YBL017C;133, YBR097W;137, YBR105C;154, YBR131W;165, YBR164C;213, YBR290W;220, YCL008C;477, YDR200C;615, YDR495C;639, YEL013W;645, YEL027W;759, YER180C-A;848, YGL104C;857, YGL124C;979, YGR141W;1008, YGR198W;1014, YGR206W;1047, YHL002W;1267, YJL036W;1378, YJR102C;1388, YJR126C;1490, YKR001C;1497, YKR014C;1499, YKR020W;1509, YKR035W-A;1617, YLR119W;1729, YLR417W;1771, YML041C;1793, YML097C;1813, YMR004W;1997, YNL093W;2075, YNL275W;2086, YNL297C;2103, YNR006W;2198, YOL129W;2247, YOR089C;2354, YPL002C;2372, YPL045W;2380, YPL065W;2532, YPR139C

Module 27

gene:15, YAL023C;38, YAR015W;52, YBL024W;54, YBL026W;69, YBL068W;79, YBL082C;104, YBR034C;138, YBR106W;159, YBR143C;170, YBR175W;194, YBR249C;196, YBR252W;201, YBR265W;210, YBR283C;247, YCR020C-A;253, YCR034W;298, YDL040C;319, YDL081C;326, YDL095W;328, YDL111C;354, YDL166C;375, YDL212W;379, YDL219W;385, YDL232W;387, YDL236W;425, YDR075W;430, YDR091C;468, YDR184C;511, YDR297W;514, YDR300C;516, YDR302W;541, YDR361C;557, YDR395W;559, YDR398W;588, YDR454C;595, YDR465C;634, YEL002C;651, YEL040W;656, YEL053C;812, YGL022W;906, YGL226C-A;1005, YGR191W;1042, YGR285C;1048, YHL003C;1073, YHR013C;1079, YHR026W;1096, YHR064C;1141, YHR170W;1167, YIL016W;1192, YIL079C;1199, YIL103W;1252, YJL002C;1305, YJL117W;1333, YJL198W;1352, YJR022W;1368, YJR069C;1369, YJR070C;1380, YJR105W;1477, YKL191W;1575, YLR017W;1629, YLR146C;1649, YLR195C;1670, YLR244C;1715, YLR372W;1760, YML019W;1761, YML022W;1799, YML106W;1807, YML125C;1861, YMR116C;1873, YMR149W;1899, YMR215W;1917, YMR246W;2017, YNL141W;2035, YNL189W;2044, YNL219C;2127, YNR046W;2196, YOL125W;2246, YOR085W;2252, YOR103C;2303, YOR253W;2304, YOR254C;2325, YOR317W;2456, YPL227C;2499, YPR051W

Module 28

gene:28, YAL051W;42, YBL002W;94, YBR009C;95, YBR010W;142, YBR112C;183, YBR231C;191, YBR245C;242, YCR004C;274, YCR084C;299, YDL042C;407, YDR032C;484, YDR223W;488, YDR227W;517, YDR310C;556, YDR392W;609, YDR485C;1028, YGR252W;1193, YIL084C;1301, YJL110C;1303, YJL115W;1365, YJR060W;1420, YKL038W;1506, YKR029C;1521, YKR064W;1539, YKR101W;1573, YLR015W;1589, YLR055C;1607, YLR103C;1771, YML041C;1925, YMR263W;1999, YNL097C;2042, YNL216W;2067, YNL261W;2098, YNL330C;2105, YNR009W;2142, YOL004W;2145, YOL012C;2322, YOR304W;2406, YPL129W;2410, YPL138C;2434, YPL181W;2483, YPR018W

Module 29

gene:22, YAL035W;48, YBL013W;125, YBR079C;372, YDL207W;430, YDR091C;441, YDR117C;574, YDR429C;614, YDR494W;679, YER025W;744, YER153C;822, YGL049C;850, YGL107C;941, YGR054W;991, YGR162W;1024, YGR246C;1315, YJL138C;1346, YJR007W;1349, YJR014W;1518, YKR059W;1648, YLR192C;1815, YMR012W;1872, YMR146C;1923, YMR260C;1947, YMR309C;1960, YNL007C;1979, YNL062C;2058, YNL244C;2150, YOL023W;2200, YOL139C;2285, YOR204W;2342, YOR361C;2400, YPL119C;2463, YPL237W;2495, YPR041W;2513, YPR086W;2541, YPR163C

Module 30

gene:77, YBL079W;324, YDL088C;332, YDL116W;472, YDR192C;529, YDR335W;585, YDR450W;720, YER105C;721, YER107C;734, YER131W;785, YFR002W;840, YGL092W;844, YGL097W;846, YGL100W;856, YGL123W;883, YGL172W;891, YGL189C;971, YGR119C;1016, YGR214W;1207, YIL115C;1268, YJL041W;1277, YJL061W;1358, YJR042W;1386, YJR123W;1425, YKL057C;1430, YKL068W;1528, YKR082W;1586, YLR048W;1677, YLR264W;1702, YLR335W;1764, YML026C;1798, YML103C;1831, YMR047C;1866, YMR129W;1874, YMR153W;1908, YMR230W;1912, YMR235C;2029, YNL178W;2089, YNL302C;2154, YOL040C;2193, YOL121C;2249, YOR098C;2272, YOR167C;2316, YOR293W

Module 31

gene:33, YAR002W;2035, YNL189W

Module 33

gene:35, YAR007C;278, YCR092C;632, YDR540C;797, YFR038W;802, YFR055W;1272, YJL051W;1325, YJL173C;1552, YLL033W;1792, YML095C;2094, YNL312W;2098, YNL330C;2178, YOL090W;2265, YOR141C;2359, YPL017C;2362, YPL022W

Module 34

gene:23, YAL036C;24, YAL038W;58, YBL039C;72, YBL072C;75, YBL076C;96, YBR017C;100, YBR025C;102, YBR031W;109, YBR048W;125, YBR079C;134, YBR101C;145, YBR118W;148, YBR121C;159, YBR143C;173, YBR189W;176, YBR196C;194, YBR249C;227, YCL030C;229, YCL037C;261, YCR053W;263, YCR059C;298, YDL040C;307, YDL055C;313, YDL069C;320, YDL082W;321,

YDL083C;350, YDL160C;383, YDL229W;398, YDR012W;403, YDR025W;430, YDR091C;446, YDR127W;450, YDR140W;473, YDR194C;475, YDR197W;480, YDR211W;514, YDR300C;538, YDR350C;554, YDR385W;557, YDR395W;567, YDR418W;600, YDR471W;616, YDR496C;624, YDR515W;657, YEL054C;679, YER025W;704, YER074W;717, YER102W;723, YER110C;744, YER153C;749, YER165W;779, YFL045C;787, YFR009W;794, YFR031C-A;817, YGL031C;822, YGL049C;834, YGL076C;895, YGL195W;914, YGL238W;917, YGL245W;941, YGR054W;955, YGR083C;962, YGR094W;991, YGR162W;1013, YGR204W;1016, YGR214W;1019, YGR222W;1021, YGR240C;1034, YGR264C;1037, YGR271W;1042, YGR285C;1046, YHL001W;1061, YHL033C;1096, YHR064C;1153, YHR203C;1157, YHR216W;1188, YIL069C;1191, YIL078W;1216, YIL133C;1311, YJL130C;1315, YJL138C;1326, YJL177W;1330, YJL191W;1346, YJR007W;1393, YJR145C;1427, YKL060C;1435, YKL081W;1471, YKL180W;1473, YKL182W;1482, YKL204W;1503, YKR026C;1518, YKR059W;1529, YKR084C;1546, YLL018C;1560, YLL045C;1584, YLR044C;1586, YLR048W;1592, YLR060W;1597, YLR075W;1631, YLR150W;1642, YLR180W;1650, YLR196W;1657, YLR215C;1672, YLR249W;1691, YLR291C;1723, YLR398C;1736, YLR432W;1741, YLR441C;1744, YLR448W;1762, YML024W;1776, YML056C;1780, YML063W;1782, YML068W;1783, YML073C;1815, YMR012W;1847, YMR080C;1861, YMR116C;1870, YMR142C;1895, YMR205C;1916, YMR242C;1921, YMR257C;1946, YMR308C;1947, YMR309C;1955, YNL001W;1963, YNL016W;2006, YNL112W;2040, YNL209W;2058, YNL244C;2088, YNL301C;2126, YNR045W;2180, YOL097C;2200, YOL139C;2234, YOR063W;2241, YOR076C;2263, YOR133W;2268, YOR151C;2306, YOR260W;2311, YOR276W;2320, YOR302W;2324, YOR312C;2333, YOR335C;2342, YOR361C;2383, YPL081W;2407, YPL131W;2423, YPL160W;2432, YPL179W;2441, YPL198W;2453, YPL220W;2455, YPL226W;2495, YPR041W;2508, YPR074C;2510, YPR080W

Module 35

gene:135, YBR102C

Module 36

gene:17, YAL026C;135, YBR102C;341, YDL137W;1580, YLR026C;1613, YLR114C;1715, YLR372W;2413, YPL145C;2482, YPR017C

Module 38

gene:873, YGL154C;2415, YPL148C;2459, YPL231W

Module 39

gene:49, YBL014C;53, YBL025W;162, YBR154C;456, YDR156W;740, YER148W;1131, YHR143W-A;1260, YJL025W;1318, YJL148W;1366, YJR063W;1449, YKL125W;1626, YLR141W;1757, YML010W;1773, YML043C;1806, YML121W;1930, YMR270C;2007, YNL113W;2061, YNL248C;2288, YOR210W;2292, YOR224C;2318, YOR295W;2335, YOR340C;2336, YOR341W;2479, YPR010C;2519, YPR110C;2553, YPR187W

Module 40

gene:46, YBL007C;276, YCR088W;351, YDL161W;1185, YIL062C;1196, YIL095W;1235, YIR006C;1387, YJR125C;1408, YKL013C;1556, YLL038C;1653, YLR206W;1964, YNL020C;1994, YNL084C

Module 41

gene:16, YAL025C;31, YAL059W;104, YBR034C;163, YBR155W;192, YBR247C;202, YBR267W;234, YCL054W;258, YCR047C;263, YCR059C;268, YCR072C;295, YDL036C;310, YDL060W;347, YDL150W;369, YDL201W;425, YDR075W;442, YDR120C;460, YDR165W;468, YDR184C;541, YDR361C;595, YDR465C;731, YER127W;763, YFL001W;829, YGL063W;835, YGL078C;994, YGR169C;1010, YGR200C;1085, YHR040W;1101, YHR070W;1146, YHR187W;1169, YIL019W;1192, YIL079C;1237, YIR012W;1242, YIR026C;1296, YJL098W;1306, YJL122W;1376, YJR097W;1444, YKL110C;1502, YKR025W;1632, YLR165C;1638, YLR175W;1718, YLR384C;1725, YLR401C;1738, YLR435W;1758, YML014W;1786, YML080W;1949, YMR312W;1979, YNL062C;1988, YNL075W;2012, YNL124W;2017, YNL141W;2079, YNL282W;2084, YNL292W;2108, YNR012W;2110, YNR015W;2127, YNR046W;2144, YOL010W;2166, YOL066C;2195, YOL124C;2196, YOL125W;2214, YOR004W;2232, YOR056C;2298, YOR243C;2317, YOR294W;2388, YPL086C;2393, YPL101W;2448, YPL212C;2534, YPR144C

Module 42

gene:589, YDR456W

Module 43

gene:294, YDL033C;369, YDL201W;751, YER168C;881, YGL169W;910, YGL232W;913, YGL236C;916, YGL243W;930, YGR024C;1146, YHR187W;1266, YJL035C;1515, YKR056W;1696, YLR316C;1725, YLR401C;1726, YLR405W;1786, YML080W;1823, YMR023C;2084, YNL292W;2110, YNR015W;2310, YOR274W

Module 44

gene:211, YBR284W;912, YGL234W;1243, YIR027C;1244, YIR028W;1245, YIR029W;1246, YIR031C;1247, YIR032C;1281, YJL070C;1380, YJR105W;1769, YML035C;2260, YOR128C

Module 47

gene:898, YGL202W;1128, YHR137W

Module 48

gene:50, YBL017C;154, YBR131W;526, YDR323C;702, YER072W;765, YFL004W;1258, YJL012C;1274, YJL053W;1295, YJL097W;1344, YJR001W;1490, YKR001C;1630, YLR148W;1743, YLR447C;2360, YPL019C;2386, YPL084W;2509, YPR079W

Module 49

gene:198, YBR257W;1085, YHR040W;1374, YJR093C;1411, YKL018W;1459, YKL149C;2047, YNL222W;2462, YPL235W

Module 50

gene:6, YAL002W;133, YBR097W;220, YCL008C;610, YDR486C;790, YFR021W;1047, YHL002W;1067, YHR001W;1400, YKL002W;1421, YKL041W;1509, YKR035W-A;1558, YLL040C;1579, YLR025W;1630, YLR148W;1643, YLR181C;1711, YLR360W;1722, YLR396C;1844, YMR077C;1892, YMR197C;1909, YMR231W;2103, YNR006W;2372, YPL045W;2401, YPL120W;2544, YPR173C

Module 51

gene:109, YBR048W;251, YCR031C;316, YDL075W;403, YDR025W;567, YDR418W;582, YDR447C;657, YEL054C;817, YGL031C;847, YGL103W;870, YGL147C;957, YGR085C;983, YGR148C;1016, YGR214W;1078, YHR021C;1111, YHR088W;1330, YJL191W;1462, YKL156W;1586, YLR048W;1597, YLR075W;1634, YLR167W;1698, YLR325C;1703, YLR340W;1727, YLR406C;1744, YLR448W;1762, YML024W;1783, YML073C;1983, YNL067W;2197, YOL127W;2234, YOR063W;2407, YPL131W;2516, YPR102C

Module 52

gene:21, YAL033W;75, YBL076C;104, YBR034C;113, YBR055C;198, YBR257W;468, YDR184C;595, YDR465C;739, YER146W;1010, YGR200C;1094, YHR062C;1146, YHR187W;1192, YIL079C;1444, YKL110C;1718, YLR384C;1738, YLR435W;1912, YMR235C;1949, YMR312W;2046, YNL221C;2079, YNL282W;2195, YOL124C;2196, YOL125W;2206, YOL149W;2388, YPL086C;2393, YPL101W

Module 53

gene:688, YER044C;803, YGL001C;807, YGL012W;944, YGR060W;999, YGR175C;1069, YHR007C;1087, YHR042W;1104, YHR072W;1148, YHR190W;1324, YJL167W;1460, YKL150W;1590, YLR056W;1605, YLR100W;1746, YLR450W;1755, YML008C;1785, YML075C;1808, YML126C;1817, YMR015C;1894, YMR202W;1897, YMR208W;1903, YMR220W;2078, YNL280C;2363, YPL028W;2398, YPL117C

Module 55

gene:1941, YMR290C

Module 56

gene:1710, YLR359W

Module 57

gene:77, YBL079W;145, YBR118W;269, YCR073W-A;324, YDL088C;332, YDL116W;472, YDR192C;529, YDR335W;720, YER105C;721, YER107C;729, YER125W;785, YFR002W;840, YGL092W;846, YGL100W;883, YGL172W;971, YGR119C;975, YGR128C;1268, YJL041W;1277, YJL061W;1358, YJR042W;1425, YKL057C;1430, YKL068W;1483, YKL205W;1528, YKR082W;1702, YLR335W;1798, YML103C;1831, YMR047C;1866, YMR129W;1874, YMR153W;2119, YNR034W;2249, YOR098C;2257, YOR112W;2510, YPR080W

Module 58

gene:33, YAR002W;77, YBL079W;202, YBR267W;557, YDR395W;590, YDR457W;672, YER009W;720, YER105C;836, YGL086W;1207, YIL115C;1528, YKR082W;1533, YKR092C;1576, YLR018C;1692, YLR293C;2249, YOR098C;2279, YOR185C

Module 59

gene:285, YDL014W;2421, YPL157W

Module 60

gene:77, YBL079W;96, YBR017C;324, YDL088C;332, YDL116W;472, YDR192C;529, YDR335W;720, YER105C;721, YER107C;785, YFR002W;840, YGL092W;846, YGL100W;883, YGL172W;971, YGR119C;1268, YJL041W;1277, YJL061W;1358, YJR042W;1425, YKL057C;1430, YKL068W;1528, YKR082W;1702, YLR335W;1798, YML103C;1831, YMR047C;1866, YMR129W;1874, YMR153W;1946, YMR308C;2249, YOR098C;2271, YOR160W

Module 62

gene:108, YBR043C;143, YBR115C;160, YBR145W;182, YBR218C;193, YBR248C;194, YBR249C;197, YBR256C;220, YCL008C;221, YCL009C;227, YCL030C;306, YDL054C;355, YDL170W;356, YDL171C;360, YDL182W;368, YDL198C;408, YDR034C;409, YDR035W;445, YDR126W;446, YDR127W;532, YDR341C;539, YDR354W;606, YDR481C;661, YEL062W;662, YEL063C;691, YER052C;694, YER055C;701, YER069W;703, YER073W;707, YER086W;711, YER090W;858, YGL125W;890, YGL184C;898, YGL202W;1035, YGR267C;1075, YHR018C;1102, YHR071W;1136, YHR162W;1282, YJL071W;1283, YJL072C;1292, YJL088W;1334, YJL200C;1350, YJR016C;1353, YJR025C;1381, YJR109C;1382, YJR110W;1383, YJR111C;1485, YKL211C;1709, YLR356W;1747, YLR451W;1820, YMR019W;1839, YMR062C;1852, YMR095C;1853, YMR096W;1957, YNL004W;2002, YNL104C;2093, YNL311C;2129, YNR050C;2160, YOL058W;2164, YOL064C;2191, YOL119C;2201, YOL140W;2255, YOR108W;2261, YOR130C;2283, YOR201C;2284, YOR202W;2291, YOR222W;2321, YOR303W;2334, YOR337W;2437, YPL188W;2467, YPL252C;2473, YPL273W;2520, YPR111W;2552, YPR185W
