# Supplementary material for: Evolution of transcriptional networks in yeast: alternative teams of transcriptional factors for different species
Source: BMC Genomics. 2016 Nov 11;17(Suppl 10):826. doi: 10.1186/s12864-016-3102-7 (PMC5123246; doi:10.1186/s12864-016-3102-7)
Supplement: Supplementary file 2 — Supplementary material: yeast species. We report the yeast species names and identifier. (PDF 16 kb) [file 12864_2016_3102_MOESM2_ESM.pdf]

Yeast species

These are species as numbered in Figure 1 and as numbered in the database.

1: Saccharomyces cerevisiae
2: Saccharomyces paradoxus
3: Saccharomyces mikatae
4. Saccharomyces bayanus
5: Candida glabrata
6: Saccharomyces castellii
7: Kluyveromyces waltii
8: Saccharomyces kluyveri
9: Kluyveromyces lactis
10: Ashbya gossypii
11: Clavispora lusitaniae
12: Debaryomyces hansenii
13: Candida guillermondii
14: Candida tropicalis
15: Candida albicans
16: Candida parapsilosis
17: Lodderomyces elongisporus
18: Yarrowia lipolytica
19: Aspergillus nidulans
20: Neurospora crassa
21: Schizosaccharomyces japonicus
22: Schizosaccharomyces octosporus
23: Schizosaccharomyces pompe
