# Supplementary material for: Evolution of transcriptional networks in yeast: alternative teams of transcriptional factors for different species
Source: BMC Genomics. 2016 Nov 11;17(Suppl 10):826. doi: 10.1186/s12864-016-3102-7 (PMC5123246; doi:10.1186/s12864-016-3102-7)
Supplement: Supplementary file 4 — Supplementary methods. We describe the method for estimating transcription factor binding probabilities for each PWM (equivalently, transcription factor) on the gene promoter, for each gene in each species. (PDF 135 kb) [file 12864_2016_3102_MOESM4_ESM.pdf]

RESEARCH METHODOLOGY

Supplementary Methods for Evolution of transcriptional networks in yeast: alternative teams of transcriptional factors for different species

Adriana Muñoz[1,2,4*], Daniella Santos Muñoz[1,2,5,6], Aleksey Zimin[1] and James A. Yorke[1,2,3]

*Correspondence:
adri.embo@gmail.com
[4]Cold Spring Harbor Laboratory, 1 Bungtown Rd., Cold Spring Harbor, NY, 11724 USA
Full list of author information is available at the end of the article

Estimating transcription factor binding probabilities

Using Positional Weight Matrices (PWMs) for 126 transcription factors available from [1] and Transfac DB [2], and the tool pwm_scan [3], we estimated the occupancy probability for each PWM (equivalently, transcription factor) on the gene promoter, for each gene in each species.

We estimated the occupancy probability of transcription factor x with w bases long binding site in the L bases long promoter of gene g as follows. We used the pwm_scan tool to find the highest scoring binding site in the promoter with score, which we denote ${S_{gx}}^{max}$, and the binding site's minimum p-value p' associated with it. Following the approach in [4], we then transform p' into a binding (or occupancy) probability $P_{x,g,s}$ in species s following [4] as follows:

$$P_{x,g,s} = (1 - p')^{L-w+1} \tag{1}$$

Then, we compute the approximately 7.4 million upstream binding probabilities for each (transcription factor, gene, species) triple; i.e., for each transcription factor (126 total) and each gene (2557 total) and each species (23 total) in our dataset.

Author details
[1]Institute for Physical Science and Technology, University of Maryland, College Park, Maryland, 20742 USA. [2]Department of Mathematics, University of Maryland, College Park, Maryland, 20742 USA. [3]Department of Physics, University of Maryland, College Park, Maryland, 20742 USA. [4]Cold Spring Harbor Laboratory, 1 Bungtown Rd., Cold Spring Harbor, NY, 11724 USA. [5]Faculty of Sciences, University of Ottawa, Ottawa, ON, K1N 6N5 Canada. [6]Faculty of Engineering, University of Ottawa, Ottawa, ON, K1N 6N5 Canada.

References

1. MacIsaac K.D., Wang, T., Gordon D.B, Gifford D.K., Stormo G.D. , and Fraenkel E. (2006) An improved map of conserved regulatory sites for Saccharomyces cerevisiae, *BMC Bioinformatics*, **7**: 113.
2. Matys, V., Kel-Margoulis, O. V., Fricke, E., Liebich, I., Land, S., *et al.* (2006) RANSFAC and its module TRANSCompel: transcriptional gene regulation in eukaryotes. *Nucleic Acids Res*, **34**, D108-110.
3. Levy S. and Hannenhalli S.(2002) Identification of transcription factor binding sites in the human genome sequence, *Mamm Genome*, **13(9)**, 510-514.
4. Chen G., Jensen ST., Stoeckert CJ Jr. (2007) Clustering of genes into regulons using integrated Modeling-COGRIM, *Genome Biology*, **8(1):**, R4 (Supplementary material).
