# Supplementary material for: Evolution of transcriptional networks in yeast: alternative teams of transcriptional factors for different species
Source: BMC Genomics. 2016 Nov 11;17(Suppl 10):826. doi: 10.1186/s12864-016-3102-7 (PMC5123246; doi:10.1186/s12864-016-3102-7)
Supplement: Supplementary file 5 — Supplementary material: the transcription factors and genes, and their skewness scores of the rewiring blocks. For each module, results are reported for its top scoring branch. Each module lists the highest scoring block. (PDF 155 kb) [file 12864_2016_3102_MOESM5_ESM.pdf]

Supplementary Material:
The transcription factors and genes, and their skewness scores of the rewiring blocks

For each module, results are reported for its top scoring branch (see Figure 1 in the main paper for the numbering of branches). Each module lists the highest scoring block. By definition a block consists of two teams of transcription factors T and T*, and a gene group. Each transcription factor and gene entry includes id, name, and skewness (rounded to 3 decimal places). For some modules no block was found.

Module 2, Branch 112, skewness score: 0.544948

T: 10,AZF1,0.349

T*: 87,SKN7,-0.269; 96,STB2,-0.262; 48,LEU3,-0.247; 115,XBP1,-0.236; 90,SNF1,-0.233; 101,STP4,-0.231; 113,UME6,-0.225; 43,IME1,-0.218; 69,PUT3,-0.218; 114,URC2,-0.212; 26,GAL4,-0.208; 1,ABF1,-0.207; 64,PDR1,-0.207; 100,STP1,-0.206; 62,NRG1,-0.206; 73,REB1,-0.203; 125,ZAP1,-0.193; 31,GCR1,-0.190; 103,SUT1,-0.186; 9,ASH1,-0.179; 121,YDR026C,-0.175; 107,TBF1,-0.175; 7,ARO80,-0.172; 80,ROX1,-0.163; 51,MBP1,-0.158; 44,INO2,-0.154; 63,OPI1,-0.153; 70,RAP1,-0.153; 23,FHL1,-0.149; 52,MCM1,-0.149; 105,SWI5,-0.147

gene: 1663,YLR229C,0.781; 900,YGL210W,0.718; 766,YFL005W,0.710; 459,YDR164C,0.708; 543,YDR363W-A,0.701; 681,YER031C,0.692; 2514,YPR095C,0.666; 1857,YMR109W,0.664; 1451,YKL129C,0.650; 1507,YKR031C,0.597; 445,YDR126W,0.587; 2074,YNL272C,0.574; 89,YBL106C,0.565; 1105,YHR073W,0.563; 2490,YPR032W,0.550; 2466,YPL249C,0.522; 40,YAR042W,0.520; 287,YDL019C,0.514; 1209,YIL118W,0.503; 911,YGL233W,0.455; 2500,YPR055W,0.435; 2297,YOR237W,0.425; 777,YFL039C,0.417; 1187,YIL068C,0.415; 2085,YNL293W,0.412; 1492,YKR003W,0.409; 1067,YHR001W,0.406; 1217,YIL138C,0.400; 2413,YPL145C,0.399; 1991,YNL079C,0.391

Module 6, Branch 112, skewness score: 0.459157

T: 78,RLR1,0.163; 49,MAC1,0.200; 94,SPT23,0.208; 10,AZF1,0.309

T*: 114,URC2,-0.324; 43,IME1,-0.300; 64,PDR1,-0.284; 103,SUT1,-0.282; 113,UME6,-0.251; 37,HAP1,-0.227; 98,STB5,-0.224; 115,XBP1,-0.222; 26,GAL4,-0.201; 87,SKN7,-0.199; 91,SNT2,-0.187; 73,REB1,-0.172

gene: 1040,YGR280C,0.554; 2286,YOR206W,0.521; 1929,YMR269W,0.400; 1151,YHR196W,0.362

Module 7, Branch 112, skewness score: 0.524301

T: 10,AZF1,0.360; 94,SPT23,0.286

T*: 96,STB2,-0.282; 90,SNF1,-0.280; 126,ATF1,-0.261; 103,SUT1,-0.258; 87,SKN7,-0.251; 51,MBP1,-0.232; 100,STP1,-0.230; 48,LEU3,-0.219; 69,PUT3,-0.218; 107,TBF1,-0.215; 1,ABF1,-0.203; 101,STP4,-0.190; 113,UME6,-0.188; 26,GAL4,-0.184; 97,STB4,-0.182; 63,OPI1,-0.179; 121,YDR026C,-0.178; 84,SFL1,-0.177; 73,REB1,-0.172; 98,STB5,-0.171; 115,XBP1,-0.167; 43,IME1,-0.157; 81,RPH1,-0.148; 9,ASH1,-0.147; 91,SNT2,-0.146

gene: 18,YAL029C,0.620; 2073,YNL271C,0.542; 153,YBR130C,0.533; 2281,YOR191W,0.517; 433,YDR097C,0.409

Module 8, Branch 112, skewness score: 0.534087

T: 10,AZF1,0.335

T*: 96,STB2,-0.306; 101,STP4,-0.261; 1,ABF1,-0.250; 48,LEU3,-0.229; 113,UME6,-0.220; 114,URC2,-0.219; 43,IME1,-0.210; 87,SKN7,-0.208; 73,REB1,-0.201; 62,NRG1,-0.196; 51,MBP1,-0.194; 103,SUT1,-0.193; 90,SNF1,-0.192; 115,XBP1,-0.189; 26,GAL4,-0.180; 7,ARO80,-0.179; 69,PUT3,-0.178; 106,SWI6,-0.177; 125,ZAP1,-0.172; 9,ASH1,-0.168; 107,TBF1,-0.168; 91,SNT2,-0.165; 37,HAP1,-0.162; 100,STP1,-0.161

gene: 1580,YLR026C,0.797; 459,YDR164C,0.751; 766,YFL005W,0.726; 335,YDL126C,0.685; 19,YAL030W,0.655; 2329,YOR327C,0.653; 618,YDR498C,0.571; 526,YDR323C,0.532; 2253,YOR106W,0.509; 2147,YOL018C,0.503; 1602,YLR093C,0.488; 597,YDR468C,0.461; 1480,YKL196C,0.460; 911,YGL233W,0.453; 2500,YPR055W,0.444; 926,YGR009C,0.442; 1187,YIL068C,0.438; 1892,YMR197C,0.417; 902,YGL212W,0.415; 1598,YLR078C,0.413; 1885,YMR183C,0.405

Module 9, Branch 114, skewness score: 0.380657

T: 51,MBP1,0.168; 106,SWI6,0.174; 115,XBP1,0.183; 44,INO2,0.195; 69,PUT3,0.195; 90,SNF1,0.206; 43,IME1,0.229; 101,STP4,0.231; 68,PHO4,0.280; 111,TYE7,0.291; 13,CBF1,0.304

T*: 77,RLM1,-0.207; 94,SPT23,-0.203; 89,SMP1,-0.202; 118,YAP5,-0.201; 24,FKH1,-0.183; 120,YAP7,-0.181; 93,SPT2,-0.176; 8,ARR1,-0.172; 67,PHO2,-0.167; 85,SFP1,-0.156; 17,CST6,-0.144; 119,YAP6,-0.144; 23,FHL1,-0.126; 52,MCM1,-0.122; 116,YAP1,-0.116; 21,DIG1,-0.114; 33,GLN3,-0.114; 25,FKH2,-0.107

gene: 52,YBL024W,0.507; 2196,YOL125W,0.484; 1979,YNL062C,0.407; 1101,YHR070W,0.348; 1308,YJL125C,0.331; 2179,YOL093W,0.317; 1753,YML005W,0.271

Module 10, Branch 114, skewness score: 0.492271

T: 100,STP1,0.162; 43,IME1,0.165; 91,SNT2,0.165; 92,SOK2,0.179; 2,ACE2,0.184; 62,NRG1,0.198; 20,DAL82,0.198; 102,SUM1,0.200; 14,CHA4,0.205; 7,ARO80,0.205; 64,PDR1,0.208; 71,RCS1,0.208; 54,MET31,0.221; 48,LEU3,0.229; 103,SUT1,0.236; 106,SWI6,0.259; 51,MBP1,0.271; 87,SKN7,0.320

T*: 50,MATA1,-0.322; 12,CAD1,-0.239

gene: 553,YDR384C,0.636; 243,YCR010C,0.599; 2102,YNR002C,0.595; 2018,YNL142W,0.449; 1241,YIR023W,0.425; 972,YGR121C,0.393; 2531,YPR138C,0.349

Module 11, Branch 112, skewness score: 0.507661

T: 10,AZF1,0.362

T*: 1,ABF1,-0.221; 125,ZAP1,-0.207; 9,ASH1,-0.186; 115,XBP1,-0.184; 48,LEU3,-0.178; 123,YOX1,-0.175; 90,SNF1,-0.169; 98,STB5,-0.159; 96,STB2,-0.154; 108,TDA9,-0.154; 26,GAL4,-0.151; 87,SKN7,-0.149; 69,PUT3,-0.147; 114,URC2,-0.140; 7,ARO80,-0.138; 58,MOT3,-0.133; 32,GCR2,-0.130; 99,STE12,-0.129; 14,CHA4,-0.123; 51,MBP1,-0.122; 126,ATF1,-0.120; 42,HSF1,-0.120; 92,SOK2,-0.120; 43,IME1,-0.117; 76,RIM101,-0.116; 62,NRG1,-0.116; 79,RME1,-0.112; 121,YDR026C,-0.112

gene: 459,YDR164C,0.724; 1722,YLR396C,0.618; 526,YDR323C,0.542; 911,YGL233W,0.491; 2500,YPR055W,0.452; 1187,YIL068C,0.436; 1909,YMR231W,0.435; 470,YDR189W,0.364

Module 12, Branch 10, skewness score: 0.509281

T: 14,CHA4,0.302; 1,ABF1,0.500

T*: 11,BAS1,-0.154; 85,SFP1,-0.143; 50,MATA1,-0.101; 6,ARG81,-0.096; 16,CRZ1,-0.079; 125,ZAP1,-0.077

gene: 2390,YPL093W,0.607; 2286,YOR206W,0.590; 392,YDR002W,0.551; 1476,YKL186C,0.476; 1358,YJR042W,0.418; 1692,YLR293C,0.413

Module 15, Branch 112, skewness score: 0.551060

T: 6,ARG81,0.249; 10,AZF1,0.265; 17,CST6,0.283; 75,RGT1,0.285; 85,SFP1,0.410; 50,MATA1,0.461

T*: 2,ACE2,-0.326; 49,MAC1,-0.312; 62,NRG1,-0.298; 14,CHA4,-0.276; 1,ABF1,-0.260; 107,TBF1,-0.255; 32,GCR2,-0.255; 7,ARO80,-0.251; 126,ATF1,-0.226; 71,RCS1,-0.221; 69,PUT3,-0.217; 100,STP1,-0.214; 31,GCR1,-0.211; 101,STP4,-0.210; 44,INO2,-0.207; 9,ASH1,-0.198; 59,MSN2,-0.195; 90,SNF1,-0.186; 89,SMP1,-0.183; 84,SFL1,-0.182; 114,URC2,-0.173; 43,IME1,-0.166; 33,GLN3,-0.165

gene: 588,YDR454C,0.581; 1769,YML035C,0.522

Module 16, Branch 112, skewness score: 0.630463

T: 10,AZF1,0.431

T*: 37,HAP1,-0.292; 113,UME6,-0.278; 26,GAL4,-0.238; 7,ARO80,-0.222; 101,STP4,-0.207; 48,LEU3,-0.204; 43,IME1,-0.198; 114,URC2,-0.195; 69,PUT3,-0.193; 90,SNF1,-0.187; 91,SNT2,-0.183; 98,STB5,-0.182; 71,RCS1,-0.175; 103,SUT1,-0.175; 62,NRG1,-0.172; 87,SKN7,-0.172; 115,XBP1,-0.164; 19,DAL81,-0.148

gene: 1647,YLR189C,0.847; 1746,YLR450W,0.755; 1897,YMR208W,0.715; 1605,YLR100W,0.715; 1785,YML075C,0.712; 1808,YML126C,0.681; 1590,YLR056W,0.624; 1324,YJL167W,0.618; 1817,YMR015C,0.616; 944,YGR060W,0.611; 1069,YHR007C,0.604; 1894,YMR202W,0.602; 1903,YMR220W,0.564; 803,YGL001C,0.496; 1104,YHR072W,0.492; 807,YGL012W,0.437

Module 17, Branch 5, skewness score: 0.488036

T: 15,CIN5,0.282; 83,RTG3,0.286; 32,GCR2,0.290; 94,SPT23,0.292; 46,IXR1,0.292; 113,UME6,0.296; 42,HSF1,0.305; 61,NDD1,0.305; 124,YRR1,0.313; 25,FKH2,0.317; 89,SMP1,0.317; 34,GTS1,0.327; 8,ARR1,0.328; 67,PHO2,0.329; 20,DAL82,0.330; 12,CAD1,0.331; 21,DIG1,0.332; 118,YAP5,0.344; 88,SKO1,0.376; 99,STE12,0.390; 30,GCN4,0.395; 36,HAC1,0.398; 79,RME1,0.408; 110,THI2,0.412; 1,ABF1,0.415; 120,YAP7,0.444; 119,YAP6,0.492; 10,AZF1,0.563

T*: 101,STP4,-0.178; 80,ROX1,-0.148; 54,MET31,-0.145; 72,RDS1,-0.120; 90,SNF1,-0.118; 55,MET32,-0.096

gene: 1482,YKL204W,0.562; 1803,YML115C,0.415

Module 20, Branch 10, skewness score: 0.575388

T: 23,FHL1,0.386; 85,SFP1,0.400; 70,RAP1,0.430

T*: 107,TBF1,-0.235; 13,CBF1,-0.142; 111,TYE7,-0.133

gene: 864,YGL135W,0.785; 2453,YPL220W,0.777; 2345,YOR369C,0.777; 1703,YLR340W,0.770; 1054,YHL015W,0.764; 2154,YOL040C,0.760; 1871,YMR143W,0.737; 321,YDL083C,0.731; 1764,YML026C,0.730; 856,YGL123W,0.719; 585,YDR450W,0.702; 1386,YJR123W,0.698; 1870,YMR142C,0.698; 320,YDL082W,0.692; 2527,YPR132W,0.688; 870,YGL147C,0.681; 1329,YJL190C,0.681; 1634,YLR167W,0.676; 657,YEL054C,0.670; 2197,YOL127W,0.669; 1168,YIL018W,0.665; 336,YDL130W,0.663; 173,YBR189W,0.663; 567,YDR418W,0.662; 970,YGR118W,0.661; 794,YFR031C-A,0.655; 2383,YPL081W,0.654; 1698,YLR325C,0.651; 1129,YHR141C,0.646; 2192,YOL120C,0.644; 1223,YIL148W,0.640; 695,YER056C-A,0.631; 1535,YKR094C,0.628; 931,YGR027C,0.624; 1046,YHL001W,0.620; 725,YER117W,0.618; 80,YBL087C,0.615; 1891,YMR194W,0.614; 1688,YLR287C-A,0.613; 2465,YPL249C-A,0.608; 1313,YJL136C,0.608; 1403,YKL006W,0.607; 2024,YNL162W,0.606; 1983,YNL067W,0.606; 816,YGL030W,0.605; 1713,YLR367W,0.598; 1061,YHL033C,0.595; 1744,YLR448W,0.593; 1471,YKL180W,0.593; 174,YBR191W,0.592; 1985,YNL069C,0.592; 1183,YIL052C,0.591; 340,YDL136W,0.591; 1586,YLR048W,0.587; 1783,YML073C,0.583; 364,YDL191W,0.583; 1326,YJL177W,0.581; 1016,YGR214W,0.581; 1216,YIL133C,0.580; 1560,YLL045C,0.575; 1597,YLR075W,0.572; 1861,YMR116C,0.569; 2382,YPL079W,0.564; 319,YDL081C,0.564; 834,YGL076C,0.564; 2389,YPL090C,0.559; 316,YDL075W,0.555; 957,YGR085C,0.553; 2441,YPL198W,0.550; 2088,YNL301C,0.547; 1701,YLR333C,0.546; 171,YBR181C,0.545; 2153,YOL039W,0.544; 2407,YPL131W,0.542; 2277,YOR182C,0.540; 1516,YKR057W,0.530;

704,YER074W,0.527; 2248,YOR096W,0.519; 1330,YJL191W,0.518; 423,YDR064W,0.510; 1188,YIL069C,0.508; 1393,YJR145C,0.506; 619,YDR500C,0.504; 2516,YPR102C,0.503; 582,YDR447C,0.503; 251,YCR031C,0.500; 1644,YLR185W,0.498; 55,YBL027W,0.497; 1153,YHR203C,0.497; 128,YBR084C-A,0.497; 1780,YML063W,0.495; 847,YGL103W,0.495; 1070,YHR010W,0.494; 1908,YMR230W,0.486; 2029,YNL178W,0.485; 1998,YNL096C,0.484; 1727,YLR406C,0.475; 933,YGR034W,0.475; 2296,YOR234C,0.474; 2412,YPL143W,0.470; 1704,YLR344W,0.468; 109,YBR048W,0.468; 600,YDR471W,0.468; 2316,YOR293W,0.467; 1762,YML024W,0.466; 1741,YLR441C,0.465; 776,YFL034C-A,0.434; 1462,YKL156W,0.430; 1593,YLR061W,0.424; 1078,YHR021C,0.420; 552,YDR382W,0.417; 84,YBL092W,0.413; 403,YDR025W,0.411; 1328,YJL189W,0.406; 311,YDL061C,0.400; 1720,YLR388W,0.397

Module 22, Branch 112, skewness score: 0.558380

T: 10,AZF1,0.378

T*: 43,IME1,-0.266; 114,URC2,-0.238; 115,XBP1,-0.217; 103,SUT1,-0.205; 69,PUT3,-0.203; 26,GAL4,-0.198; 37,HAP1,-0.196; 101,STP4,-0.184; 87,SKN7,-0.173; 113,UME6,-0.172; 96,STB2,-0.166; 100,STP1,-0.163; 9,ASH1,-0.163; 105,SWI5,-0.160; 14,CHA4,-0.155; 64,PDR1,-0.154; 48,LEU3,-0.153; 90,SNF1,-0.150; 44,INO2,-0.145; 7,ARO80,-0.137

gene: 1076,YHR019C,0.757; 1987,YNL073W,0.729; 1191,YIL078W,0.683; 708,YER087W,0.680; 402,YDR023W,0.672; 1077,YHR020W,0.658; 1717,YLR382C,0.647; 1003,YGR185C,0.640; 2497,YPR047W,0.637; 2391,YPL097W,0.588; 962,YGR094W,0.582; 772,YFL022C,0.546; 1034,YGR264C,0.538; 148,YBR121C,0.537; 2511,YPR081C,0.510; 2423,YPL160W,0.472; 410,YDR037W,0.457; 2180,YOL097C,0.426; 248,YCR024C,0.424; 504,YDR268W,0.420; 1546,YLL018C,0.417; 1114,YHR091C,0.416; 532,YDR341C,0.406

Module 23, Branch 112, skewness score: 0.495321

T: 89,SMP1,0.200; 61,NDD1,0.292; 10,AZF1,0.386

T*: 43,IME1,-0.289; 96,STB2,-0.278; 101,STP4,-0.276; 26,GAL4,-0.264; 113,UME6,-0.245; 7,ARO80,-0.245; 64,PDR1,-0.242; 37,HAP1,-0.227; 103,SUT1,-0.219; 97,STB4,-0.198; 48,LEU3,-0.195; 114,URC2,-0.191; 107,TBF1,-0.190; 69,PUT3,-0.189; 91,SNT2,-0.187; 90,SNF1,-0.185; 62,NRG1,-0.183; 73,REB1,-0.179; 108,TDA9,-0.176; 87,SKN7,-0.171; 92,SOK2,-0.168; 100,STP1,-0.163; 121,YDR026C,-0.160; 9,ASH1,-0.152; 105,SWI5,-0.150; 75,RGT1,-0.147

gene: 258,YCR047C,0.651; 2530,YPR137W,0.539; 731,YER127W,0.446; 1651,YLR197W,0.431; 2470,YPL266W,0.409

Module 24, Branch 112, skewness score: 0.451519

T: 61,NDD1,0.224; 10,AZF1,0.367

T*: 114,URC2,-0.209; 96,STB2,-0.205; 26,GAL4,-0.177; 73,REB1,-0.176; 90,SNF1,-0.174; 48,LEU3,-0.171; 113,UME6,-0.165; 87,SKN7,-0.163; 105,SWI5,-0.161; 43,IME1,-0.160; 115,XBP1,-0.158; 100,STP1,-0.156; 7,ARO80,-0.156; 1,ABF1,-0.155; 37,HAP1,-0.154; 103,SUT1,-0.152; 9,ASH1,-0.150; 101,STP4,-0.149; 97,STB4,-0.143; 91,SNT2,-0.138; 44,INO2,-0.134; 80,ROX1,-0.117; 62,NRG1,-0.110; 121,YDR026C,-0.105

gene: 1017,YGR218W,0.628; 529,YDR335W,0.585; 1358,YJR042W,0.563; 883,YGL172W,0.560; 2249,YOR098C,0.557; 1425,YKL057C,0.528; 2035,YNL189W,0.528; 1268,YJL041W,0.506; 1874,YMR153W,0.479; 1798,YML103C,0.465; 785,YFR002W,0.458; 1866,YMR129W,0.454; 914,YGL238W,0.434; 324,YDL088C,0.432; 1528,YKR082W,0.417; 332,YDL116W,0.402; 33,YAR002W,0.400; 720,YER105C,0.379; 77,YBL079W,0.371; 457,YDR159W,0.363; 1702,YLR335W,0.354; 919,YGL247W,0.332; 971,YGR119C,0.325; 1082,YHR036W,0.318

Module 25, Branch 112, skewness score: 0.434986

T: 24,FKH1,0.220; 94,SPT23,0.223; 25,FKH2,0.248; 10,AZF1,0.356

T*: 87,SKN7,-0.231; 43,IME1,-0.218; 114,URC2,-0.215; 101,STP4,-0.202; 26,GAL4,-0.198; 96,STB2,-0.194; 100,STP1,-0.189; 113,UME6,-0.188; 48,LEU3,-0.183; 115,XBP1,-0.182; 7,ARO80,-0.178; 91,SNT2,-0.178; 69,PUT3,-0.177; 103,SUT1,-0.159; 73,REB1,-0.157; 62,NRG1,-0.157; 97,STB4,-0.151; 80,ROX1,-0.147; 51,MBP1,-0.145; 90,SNF1,-0.140; 37,HAP1,-0.136; 64,PDR1,-0.129; 121,YDR026C,-0.128

gene: 1179,YIL045W,0.629; 288,YDL020C,0.626; 2175,YOL082W,0.590; 1205,YIL112W,0.585; 1647,YLR189C,0.579; 1512,YKR042W,0.569; 1876,YMR159C,0.564; 2253,YOR106W,0.541; 889,YGL180W,0.508; 1579,YLR025W,0.507; 937,YGR043C,0.477; 1640,YLR178C,0.464; 875,YGL156W,0.460; 1289,YJL083W,0.457; 1202,YIL107C,0.456; 1323,YJL164C,0.452; 1203,YIL109C,0.441; 497,YDR255C,0.440; 2104,YNR007C,0.434; 2070,YNL265C,0.430; 1498,YKR019C,0.428; 528,YDR330W,0.423; 939,YGR046W,0.416; 2181,YOL100W,0.414; 154,YBR131W,0.395; 415,YDR043C,0.395; 1854,YMR097C,0.388; 747,YER162C,0.382; 804,YGL006W,0.381; 1732,YLR423C,0.381; 1320,YJL155C,0.379; 2549,YPR181C,0.370; 790,YFR021W,0.367;

879,YGL166W,0.366; 1327,YJL178C,0.357; 421,YDR059C,0.357; 1267,YJL036W,0.335; 2103,YNR006W,0.334; 857,YGL124C,0.332; 208,YBR280C,0.332; 572,YDR425W,0.331; 2262,YOR132W,0.328; 638,YEL012W,0.306

Module 27, Branch 112, skewness score: 0.395367

T: 25,FKH2,0.163; 24,FKH1,0.166; 10,AZF1,0.325

T*: 114,URC2,-0.244; 26,GAL4,-0.226; 43,IME1,-0.220; 101,STP4,-0.215; 87,SKN7,-0.215; 113,UME6,-0.198; 48,LEU3,-0.198; 90,SNF1,-0.197; 115,XBP1,-0.195; 103,SUT1,-0.193; 37,HAP1,-0.192; 7,ARO80,-0.181; 100,STP1,-0.179; 69,PUT3,-0.172; 80,ROX1,-0.166; 96,STB2,-0.156; 14,CHA4,-0.156; 1,ABF1,-0.156; 97,STB4,-0.151; 91,SNT2,-0.147; 73,REB1,-0.146; 63,OPI1,-0.142; 62,NRG1,-0.133; 121,YDR026C,-0.130; 64,PDR1,-0.124

gene: 1917,YMR246W,0.570; 2325,YOR317W,0.556; 138,YBR106W,0.522; 812,YGL022W,0.501; 1761,YML022W,0.478; 328,YDL111C,0.475; 1079,YHR026W,0.473; 1096,YHR064C,0.466; 196,YBR252W,0.463; 1252,YJL002C,0.451; 2035,YNL189W,0.451; 511,YDR297W,0.448; 54,YBL026W,0.443; 159,YBR143C,0.443; 1807,YML125C,0.442; 425,YDR075W,0.432; 1575,YLR017W,0.426; 1141,YHR170W,0.425; 104,YBR034C,0.415; 557,YDR395W,0.413; 2304,YOR254C,0.410; 2499,YPR051W,0.406; 1048,YHL003C,0.396; 1380,YJR105W,0.396; 1670,YLR244C,0.394; 1333,YJL198W,0.380; 1042,YGR285C,0.378; 1073,YHR013C,0.373; 194,YBR249C,0.364; 1649,YLR195C,0.359; 2246,YOR085W,0.358; 52,YBL024W,0.352; 1629,YLR146C,0.347; 1305,YJL117W,0.345; 1760,YML019W,0.344; 1861,YMR116C,0.336; 1167,YIL016W,0.334; 15,YAL023C,0.332; 354,YDL166C,0.327; 1477,YKL191W,0.326; 326,YDL095W,0.325; 595,YDR465C,0.321; 1005,YGR191W,0.321; 2044,YNL219C,0.321; 1873,YMR149W,0.318; 210,YBR283C,0.316; 651,YEL040W,0.310; 2127,YNR046W,0.302; 247,YCR020C-A,0.290

Module 28, Branch 112, skewness score: 0.366731

T: 25,FKH2,0.154; 61,NDD1,0.186; 89,SMP1,0.188; 93,SPT2,0.199; 77,RLM1,0.215; 10,AZF1,0.298

T*: 113,UME6,-0.316; 101,STP4,-0.316; 103,SUT1,-0.284; 114,URC2,-0.268; 43,IME1,-0.256; 9,ASH1,-0.251; 26,GAL4,-0.249; 100,STP1,-0.248; 69,PUT3,-0.247; 7,ARO80,-0.243; 90,SNF1,-0.238; 96,STB2,-0.235; 73,REB1,-0.227; 37,HAP1,-0.218; 87,SKN7,-0.218; 121,YDR026C,-0.191; 98,STB5,-0.191; 51,MBP1,-0.188; 48,LEU3,-

0.185; 91,SNT2,-0.184; 64,PDR1,-0.178; 97,STB4,-0.178; 14,CHA4,-0.171; 104,SWI4,-0.164; 80,ROX1,-0.162; 1,ABF1,-0.161; 106,SWI6,-0.159

gene: 42,YBL002W,0.622; 94,YBR009C,0.563; 1301,YJL110C,0.511; 2322,YOR304W,0.478; 1521,YKR064W,0.467; 1589,YLR055C,0.456; 1771,YML041C,0.454; 95,YBR010W,0.436; 142,YBR112C,0.419; 2142,YOL004W,0.412; 1303,YJL115W,0.393; 2042,YNL216W,0.379; 2145,YOL012C,0.371; 484,YDR223W,0.370; 1999,YNL097C,0.354; 2410,YPL138C,0.333; 2406,YPL129W,0.332; 556,YDR392W,0.324

Module 29, Branch 10, skewness score: 0.399160

T: 96,STB2,0.307; 73,REB1,0.325; 1,ABF1,0.418

T*: 50,MATA1,-0.110; 20,DAL82,-0.086; 57,MIG1,-0.076

gene: 2541,YPR163C,0.530; 1346,YJR007W,0.528; 1315,YJL138C,0.522; 2285,YOR204W,0.519; 2400,YPL119C,0.504; 2058,YNL244C,0.483; 1518,YKR059W,0.482; 125,YBR079C,0.447; 2463,YPL237W,0.375; 2342,YOR361C,0.361; 941,YGR054W,0.356; 2200,YOL139C,0.314; 2495,YPR041W,0.312

Module 30, Branch 112, skewness score: 0.564367

T: 61,NDD1,0.172; 10,AZF1,0.328

T*: 43,IME1,-0.237; 90,SNF1,-0.235; 101,STP4,-0.230; 105,SWI5,-0.228; 87,SKN7,-0.226; 114,URC2,-0.225; 26,GAL4,-0.224; 73,REB1,-0.221; 48,LEU3,-0.221; 96,STB2,-0.209; 9,ASH1,-0.208; 7,ARO80,-0.202; 1,ABF1,-0.196; 103,SUT1,-0.196; 113,UME6,-0.187; 70,RAP1,-0.182; 98,STB5,-0.180; 37,HAP1,-0.175; 115,XBP1,-0.174; 100,STP1,-0.169; 97,STB4,-0.169; 64,PDR1,-0.165; 86,SIP4,-0.159; 62,NRG1,-0.150; 51,MBP1,-0.148; 2,ACE2,-0.148; 44,INO2,-0.148; 69,PUT3,-0.140; 121,YDR026C,-0.138; 36,HAC1,-0.135; 23,FHL1,-0.133; 91,SNT2,-0.133; 63,OPI1,-0.129; 102,SUM1,-0.128; 59,MSN2,-0.124

gene: 529,YDR335W,0.564; 2249,YOR098C,0.531; 1358,YJR042W,0.525; 1425,YKL057C,0.522; 2154,YOL040C,0.521; 883,YGL172W,0.512; 1268,YJL041W,0.464; 585,YDR450W,0.452; 1874,YMR153W,0.449; 785,YFR002W,0.447; 1016,YGR214W,0.445; 1798,YML103C,0.439; 1764,YML026C,0.439; 1586,YLR048W,0.437; 1866,YMR129W,0.434; 1528,YKR082W,0.412; 324,YDL088C,0.404; 2029,YNL178W,0.370; 332,YDL116W,0.367; 856,YGL123W,0.351; 1702,YLR335W,0.340; 971,YGR119C,0.340; 720,YER105C,0.339; 77,YBL079W,0.334; 846,YGL100W,0.293

Module 31, Branch 112, skewness score: 0.600039

T: 89,SMP1,0.265; 10,AZF1,0.495

T*: 114,URC2,-0.321; 26,GAL4,-0.266; 113,UME6,-0.251; 103,SUT1,-0.244; 12,CAD1,-0.239; 1,ABF1,-0.231; 46,IXR1,-0.230; 37,HAP1,-0.228; 69,PUT3,-0.226; 43,IME1,-0.224; 90,SNF1,-0.219; 48,LEU3,-0.212; 73,REB1,-0.208; 51,MBP1,-0.191; 97,STB4,-0.186; 87,SKN7,-0.184; 9,ASH1,-0.182; 96,STB2,-0.178; 108,TDA9,-0.167

gene: 2035,YNL189W,0.677; 33,YAR002W,0.523

Module 34, Branch 10, skewness score: 0.573246

T: 23,FHL1,0.394; 85,SFP1,0.409; 70,RAP1,0.448

T*: 107,TBF1,-0.225; 111,TYE7,-0.122; 13,CBF1,-0.122

gene: 2453,YPL220W,0.777; 321,YDL083C,0.731; 1870,YMR142C,0.698; 320,YDL082W,0.692; 657,YEL054C,0.670; 173,YBR189W,0.663; 567,YDR418W,0.662; 794,YFR031C-A,0.655; 2383,YPL081W,0.654; 1046,YHL001W,0.620; 1061,YHL033C,0.595; 1744,YLR448W,0.593; 1471,YKL180W,0.593; 1586,YLR048W,0.587; 1783,YML073C,0.583; 1326,YJL177W,0.581; 1016,YGR214W,0.581; 1216,YIL133C,0.580; 1560,YLL045C,0.575; 1597,YLR075W,0.572; 145,YBR118W,0.571; 1861,YMR116C,0.569; 834,YGL076C,0.564; 2441,YPL198W,0.550; 2088,YNL301C,0.547; 2510,YPR080W,0.543; 2407,YPL131W,0.542; 704,YER074W,0.527; 1330,YJL191W,0.518; 1188,YIL069C,0.508; 1393,YJR145C,0.506; 1153,YHR203C,0.497; 1780,YML063W,0.495; 229,YCL037C,0.479; 109,YBR048W,0.468; 600,YDR471W,0.468; 1762,YML024W,0.466; 1741,YLR441C,0.465; 403,YDR025W,0.411

Module 35, Branch 4, skewness score: 0.598101

T: 47,JHD1,0.206; 49,MAC1,0.212; 77,RLM1,0.220; 21,DIG1,0.223; 93,SPT2,0.234; 33,GLN3,0.254; 109,TEC1,0.269; 75,RGT1,0.271; 89,SMP1,0.287; 24,FKH1,0.294; 64,PDR1,0.296; 120,YAP7,0.298; 37,HAP1,0.308; 25,FKH2,0.311; 12,CAD1,0.366; 10,AZF1,0.382; 61,NDD1,0.383

T*: 108,TDA9,-0.499; 4,AFT2,-0.383; 71,RCS1,-0.334; 105,SWI5,-0.296; 98,STB5,-0.292; 113,UME6,-0.282; 63,OPI1,-0.276; 44,INO2,-0.273; 31,GCR1,-0.263; 38,HAP2,-0.253

gene: 135,YBR102C,0.598

Module 36, Branch 112, skewness score: 0.516735

T: 119,YAP6,0.224; 10,AZF1,0.375

T*: 87,SKN7,-0.321; 43,IME1,-0.312; 114,URC2,-0.289; 113,UME6,-0.281; 37,HAP1,-0.263; 96,STB2,-0.258; 69,PUT3,-0.255; 90,SNF1,-0.247; 91,SNT2,-0.233; 48,LEU3,-0.231; 60,MSN4,-0.214; 84,SFL1,-0.207; 73,REB1,-0.200; 101,STP4,-0.196; 103,SUT1,-0.190; 9,ASH1,-0.189; 22,ECM22,-0.183; 59,MSN2,-0.180; 100,STP1,-0.174; 70,RAP1,-0.173; 14,CHA4,-0.172; 7,ARO80,-0.171; 80,ROX1,-0.167; 115,XBP1,-0.165; 26,GAL4,-0.164

gene: 1580,YLR026C,0.645; 17,YAL026C,0.546; 1613,YLR114C,0.534; 341,YDL137W,0.486; 2413,YPL145C,0.372

Module 39, Branch 10, skewness score: 0.566253

T: 1,ABF1,0.442

T*: 95,STB1,-0.184; 94,SPT23,-0.138; 109,TEC1,-0.132; 26,GAL4,-0.121; 103,SUT1,-0.111; 104,SWI4,-0.109; 57,MIG1,-0.103; 4,AFT2,-0.097

gene: 2553,YPR187W,0.694; 740,YER148W,0.643; 2288,YOR210W,0.620; 2519,YPR110C,0.608; 1366,YJR063W,0.545; 2007,YNL113W,0.544; 1757,YML010W,0.527; 2479,YPR010C,0.349

Module 40, Branch 20, skewness score: 0.698884

T: 6,ARG81,0.308; 104,SWI4,0.317; 110,THI2,0.318; 64,PDR1,0.328; 10,AZF1,0.329; 2,ACE2,0.329; 111,TYE7,0.333; 85,SFP1,0.333; 82,RPN4,0.339; 97,STB4,0.344; 81,RPH1,0.346; 51,MBP1,0.359; 38,HAP2,0.377; 52,MCM1,0.386; 86,SIP4,0.391; 80,ROX1,0.397; 23,FHL1,0.398; 96,STB2,0.398; 22,ECM22,0.429; 14,CHA4,0.455; 62,NRG1,0.471; 87,SKN7,0.480; 103,SUT1,0.483; 70,RAP1,0.487; 107,TBF1,0.507; 105,SWI5,0.527; 92,SOK2,0.529; 50,MATA1,0.535; 95,STB1,0.547; 60,MSN4,0.567; 115,XBP1,0.568; 69,PUT3,0.593; 59,MSN2,0.611

T*: 120,YAP7,-0.281; 29,GAT3,-0.261

gene: 351,YDL161W,0.708; 1556,YLL038C,0.699; 1653,YLR206W,0.690

Module 41, Branch 112, skewness score: 0.458146

T: 61,NDD1,0.224; 10,AZF1,0.366

T*: 96,STB2,-0.223; 26,GAL4,-0.217; 101,STP4,-0.202; 48,LEU3,-0.185; 7,ARO80,-0.182; 107,TBF1,-0.181; 1,ABF1,-0.181; 37,HAP1,-0.165; 114,URC2,-0.165; 43,IME1,-0.164; 103,SUT1,-0.159; 14,CHA4,-0.158; 80,ROX1,-0.156; 113,UME6,-0.155; 69,PUT3,-0.153; 73,REB1,-0.153; 125,ZAP1,-0.149; 90,SNF1,-0.148; 115,XBP1,-0.144; 87,SKN7,-0.140; 86,SIP4,-0.125; 92,SOK2,-0.123; 62,NRG1,-0.117

gene: 258,YCR047C,0.614; 2534,YPR144C,0.608; 1725,YLR401C,0.600; 2393,YPL101W,0.599; 1376,YJR097W,0.593; 2317,YOR294W,0.554; 1296,YJL098W,0.523; 2232,YOR056C,0.511; 2144,YOL010W,0.508; 1237,YIR012W,0.500; 347,YDL150W,0.492; 2110,YNR015W,0.478; 731,YER127W,0.472; 234,YCL054W,0.459; 1638,YLR175W,0.458; 268,YCR072C,0.446; 1192,YIL079C,0.406; 2127,YNR046W,0.398; 1146,YHR187W,0.394; 1085,YHR040W,0.391; 460,YDR165W,0.383; 2195,YOL124C,0.377; 1502,YKR025W,0.368; 1718,YLR384C,0.356; 104,YBR034C,0.349; 2079,YNL282W,0.338; 835,YGL078C,0.334; 202,YBR267W,0.320

Module 42, Branch 113, skewness score: 0.654650

T: 64,PDR1,0.234; 28,GAT1,0.239; 106,SWI6,0.258; 42,HSF1,0.291; 56,MET4,0.294; 90,SNF1,0.316; 55,MET32,0.370; 26,GAL4,0.374; 86,SIP4,0.455

T*: 14,CHA4,-0.513; 119,YAP6,-0.367; 62,NRG1,-0.312; 59,MSN2,-0.297; 118,YAP5,-0.291; 60,MSN4,-0.261

gene: 589,YDR456W,0.655

Module 43, Branch 112, skewness score: 0.487633

T: 61,NDD1,0.235; 94,SPT23,0.278; 10,AZF1,0.411

T*: 7,ARO80,-0.256; 101,STP4,-0.255; 113,UME6,-0.245; 80,ROX1,-0.205; 1,ABF1,-0.201; 44,INO2,-0.189; 48,LEU3,-0.181; 91,SNT2,-0.174; 100,STP1,-0.172; 87,SKN7,-0.166; 43,IME1,-0.164; 37,HAP1,-0.163; 90,SNF1,-0.161; 114,URC2,-0.161; 96,STB2,-0.159; 63,OPI1,-0.143; 13,CBF1,-0.142; 26,GAL4,-0.140; 23,FHL1,-0.138

gene: 1725,YLR401C,0.680; 930,YGR024C,0.577; 1266,YJL035C,0.546; 2110,YNR015W,0.501; 1146,YHR187W,0.374; 294,YDL033C,0.369; 2310,YOR274W,0.367

Module 44, Branch 4, skewness score: 0.477446

T: 33,GLN3,0.262; 43,IME1,0.263; 20,DAL82,0.265; 89,SMP1,0.274; 10,AZF1,0.275; 77,RLM1,0.280; 29,GAT3,0.281; 46,IXR1,0.281; 49,MAC1,0.292; 52,MCM1,0.293; 91,SNT2,0.302; 7,ARO80,0.303; 107,TBF1,0.306; 75,RGT1,0.307; 25,FKH2,0.310; 60,MSN4,0.312; 59,MSN2,0.313; 64,PDR1,0.326; 79,RME1,0.336; 61,NDD1,0.343; 113,UME6,0.369; 1,ABF1,0.522

T*: 84,SFL1,-0.168

gene: 211,YBR284W,0.645; 1281,YJL070C,0.594; 1769,YML035C,0.521; 1247,YIR032C,0.499; 912,YGL234W,0.386; 1244,YIR028W,0.373; 1246,YIR031C,0.324

Module 47, Branch 112, skewness score: 0.539219

T: 75,RGT1,0.160; 20,DAL82,0.165; 77,RLM1,0.175; 17,CST6,0.176; 59,MSN2,0.180; 118,YAP5,0.182; 25,FKH2,0.184; 107,TBF1,0.197; 21,DIG1,0.201; 99,STE12,0.213; 94,SPT23,0.217; 28,GAT1,0.249; 10,AZF1,0.254; 120,YAP7,0.254; 12,CAD1,0.255; 93,SPT2,0.259; 78,RLR1,0.270; 24,FKH1,0.283; 110,THI2,0.316

T*: 113,UME6,-0.438; 114,URC2,-0.406; 90,SNF1,-0.389; 87,SKN7,-0.387; 102,SUM1,-0.372; 26,GAL4,-0.330; 101,STP4,-0.292; 64,PDR1,-0.276; 103,SUT1,-0.267; 65,PDR3,-0.265; 43,IME1,-0.256; 80,ROX1,-0.233; 86,SIP4,-0.232

gene: 1128,YHR137W,0.546; 898,YGL202W,0.533

Module 48, Branch 112, skewness score: 0.467428

T: 94,SPT23,0.223; 10,AZF1,0.415

T*: 114,URC2,-0.208; 26,GAL4,-0.190; 96,STB2,-0.178; 90,SNF1,-0.177; 69,PUT3,-0.168; 103,SUT1,-0.167; 87,SKN7,-0.163; 43,IME1,-0.160; 7,ARO80,-0.150; 97,STB4,-0.149; 101,STP4,-0.148; 48,LEU3,-0.144; 14,CHA4,-0.144; 115,XBP1,-0.129; 73,REB1,-0.125; 19,DAL81,-0.123; 37,HAP1,-0.116; 98,STB5,-0.114; 62,NRG1,-0.112; 76,RIM101,-0.109

gene: 1490,YKR001C,0.669; 154,YBR131W,0.590; 1274,YJL053W,0.501; 50,YBL017C,0.486; 702,YER072W,0.481; 2360,YPL019C,0.447; 765,YFL004W,0.421; 1295,YJL097W,0.408; 1344,YJR001W,0.403; 1258,YJL012C,0.384; 526,YDR323C,0.352

Module 49, Branch 112, skewness score: 0.395851

T: 10,AZF1,0.431

T*: 14,CHA4,-0.170; 96,STB2,-0.168; 48,LEU3,-0.148; 105,SWI5,-0.148; 98,STB5,-0.148; 84,SFL1,-0.146; 7,ARO80,-0.134; 114,URC2,-0.132; 87,SKN7,-0.130; 23,FHL1,-0.129; 26,GAL4,-0.125; 107,TBF1,-0.116; 43,IME1,-0.107; 73,REB1,-0.101; 9,ASH1,-0.100; 70,RAP1,-0.099; 81,RPH1,-0.095; 97,STB4,-0.092; 37,HAP1,-0.089; 100,STP1,-0.088; 58,MOT3,-0.086

gene: 2047,YNL222W,0.787; 2462,YPL235W,0.693; 1085,YHR040W,0.509; 1411,YKL018W,0.493; 1374,YJR093C,0.422; 1459,YKL149C,0.409

Module 50, Branch 4, skewness score: 0.392003

T: 12,CAD1,0.175; 77,RLM1,0.198; 120,YAP7,0.204; 24,FKH1,0.224; 61,NDD1,0.239; 25,FKH2,0.252; 21,DIG1,0.264; 89,SMP1,0.284; 10,AZF1,0.339

T*: 87,SKN7,-0.214; 2,ACE2,-0.199; 103,SUT1,-0.189; 101,STP4,-0.166; 14,CHA4,-0.161; 91,SNT2,-0.157; 90,SNF1,-0.150; 83,RTG3,-0.141; 9,ASH1,-0.139; 26,GAL4,-0.136; 40,HAP4,-0.135; 80,ROX1,-0.134; 97,STB4,-0.133; 38,HAP2,-0.118; 70,RAP1,-0.115; 59,MSN2,-0.114

gene: 1711,YLR360W,0.482; 1892,YMR197C,0.438; 6,YAL002W,0.427; 2372,YPL045W,0.405; 610,YDR486C,0.349; 1421,YKL041W,0.331; 133,YBR097W,0.311

Module 51, Branch 10, skewness score: 0.733706

T: 23,FHL1,0.398; 85,SFP1,0.400; 70,RAP1,0.464

T*: 107,TBF1,-0.313

gene: 870,YGL147C,0.991; 1983,YNL067W,0.900; 1698,YLR325C,0.853; 567,YDR418W,0.846; 657,YEL054C,0.828; 1634,YLR167W,0.813; 847,YGL103W,0.774; 1016,YGR214W,0.768; 1586,YLR048W,0.763; 1703,YLR340W,0.762; 2516,YPR102C,0.671; 957,YGR085C,0.667; 1783,YML073C,0.659; 2197,YOL127W,0.654; 1597,YLR075W,0.654; 1744,YLR448W,0.653; 2407,YPL131W,0.614; 316,YDL075W,0.552; 1330,YJL191W,0.517

Module 52, Branch 4, skewness score: 0.642055

T: 96,STB2,0.419; 73,REB1,0.518

T*: 40,HAP4,-0.258; 108,TDA9,-0.173; 85,SFP1,-0.158; 70,RAP1,-0.157; 44,INO2,-0.149; 62,NRG1,-0.146

gene: 2195,YOL124C,0.862; 104,YBR034C,0.862; 75,YBL076C,0.853; 2046,YNL221C,0.726; 595,YDR465C,0.541; 2196,YOL125W,0.355; 2206,YOL149W,0.294

Module 53, Branch 112, skewness score: 0.583221

T: 10,AZF1,0.401

T*: 113,UME6,-0.267; 37,HAP1,-0.255; 26,GAL4,-0.244; 43,IME1,-0.228; 7,ARO80,-0.210; 69,PUT3,-0.203; 101,STP4,-0.199; 114,URC2,-0.193; 103,SUT1,-0.178; 48,LEU3,-0.174; 80,ROX1,-0.162; 62,NRG1,-0.160; 87,SKN7,-0.159; 90,SNF1,-0.158; 91,SNT2,-0.156; 115,XBP1,-0.155; 98,STB5,-0.154; 71,RCS1,-0.148; 100,STP1,-0.147; 64,PDR1,-0.147; 19,DAL81,-0.133

gene: 1746,YLR450W,0.755; 1785,YML075C,0.713; 1897,YMR208W,0.708; 1087,YHR042W,0.701; 1605,YLR100W,0.689; 1808,YML126C,0.688; 1590,YLR056W,0.608; 1817,YMR015C,0.604; 1324,YJL167W,0.595; 944,YGR060W,0.590; 1894,YMR202W,0.582; 2363,YPL028W,0.582; 1069,YHR007C,0.578; 1903,YMR220W,0.557; 999,YGR175C,0.502; 1104,YHR072W,0.490; 803,YGL001C,0.487; 807,YGL012W,0.443; 1460,YKL150W,0.415; 688,YER044C,0.376

Module 55, Branch 4, skewness score: 0.726213

T: 48,LEU3,0.339; 114,URC2,0.339; 64,PDR1,0.393; 43,IME1,0.409; 89,SMP1,0.414; 103,SUT1,0.418; 52,MCM1,0.420; 101,STP4,0.542; 10,AZF1,0.575; 113,UME6,0.619; 96,STB2,0.648; 73,REB1,0.650

T*: 54,MET31,-0.324; 108,TDA9,-0.322; 99,STE12,-0.311; 55,MET32,-0.287; 86,SIP4,-0.283; 125,ZAP1,-0.267; 6,ARG81,-0.247; 29,GAT3,-0.245; 11,BAS1,-0.232; 47,JHD1,-0.221; 31,GCR1,-0.194; 34,GTS1,-0.172; 28,GAT1,-0.169; 40,HAP4,-0.167

gene: 1941,YMR290C,0.726

Module 56, Branch 113, skewness score: 0.696852

T: 37,HAP1,0.270; 30,GCN4,0.281; 82,RPN4,0.293; 71,RCS1,0.293; 83,RTG3,0.309; 85,SFP1,0.316; 70,RAP1,0.335; 123,YOX1,0.376; 9,ASH1,0.436; 23,FHL1,0.490; 87,SKN7,0.500; 91,SNT2,0.531

T*: 89,SMP1,-0.456; 78,RLR1,-0.322; 32,GCR2,-0.296; 51,MBP1,-0.236

gene: 1710,YLR359W,0.697

Module 57, Branch 112, skewness score: 0.467261

T: 61,NDD1,0.235; 10,AZF1,0.378

T*: 115,XBP1,-0.225; 101,STP4,-0.205; 90,SNF1,-0.187; 48,LEU3,-0.187; 114,URC2,-0.185; 43,IME1,-0.180; 1,ABF1,-0.179; 7,ARO80,-0.172; 113,UME6,-0.168; 105,SWI5,-0.168; 26,GAL4,-0.163; 103,SUT1,-0.163; 37,HAP1,-0.161; 73,REB1,-0.158; 96,STB2,-0.158; 87,SKN7,-0.157; 100,STP1,-0.155; 82,RPN4,-0.152; 91,SNT2,-0.139; 97,STB4,-0.135; 126,ATF1,-0.133; 44,INO2,-0.132; 62,NRG1,-0.130; 9,ASH1,-0.115; 2,ACE2,-0.115

gene: 529,YDR335W,0.595; 883,YGL172W,0.566; 269,YCR073W-A,0.558; 2249,YOR098C,0.556; 2119,YNR034W,0.548; 1358,YJR042W,0.543; 1425,YKL057C,0.534; 1483,YKL205W,0.530; 145,YBR118W,0.509; 2510,YPR080W,0.504; 1268,YJL041W,0.497; 1874,YMR153W,0.479; 1798,YML103C,0.470; 1866,YMR129W,0.450; 785,YFR002W,0.445; 2257,YOR112W,0.437; 729,YER125W,0.434; 324,YDL088C,0.432; 1528,YKR082W,0.403; 332,YDL116W,0.399; 975,YGR128C,0.387; 720,YER105C,0.379; 77,YBL079W,0.371; 1702,YLR335W,0.337; 971,YGR119C,0.317

Module 58, Branch 112, skewness score: 0.542402

T: 10,AZF1,0.362

T*: 26,GAL4,-0.244; 96,STB2,-0.240; 87,SKN7,-0.229; 90,SNF1,-0.226; 37,HAP1,-0.224; 115,XBP1,-0.203; 97,STB4,-0.197; 114,URC2,-0.194; 69,PUT3,-0.189; 100,STP1,-0.188; 113,UME6,-0.187; 103,SUT1,-0.180; 48,LEU3,-0.179; 43,IME1,-0.178; 9,ASH1,-0.177; 73,REB1,-0.175; 80,ROX1,-0.163; 75,RGT1,-0.162; 105,SWI5,-0.161; 101,STP4,-0.152; 19,DAL81,-0.143; 108,TDA9,-0.130; 7,ARO80,-0.128; 65,PDR3,-0.128; 98,STB5,-0.124

gene: 2249,YOR098C,0.694; 590,YDR457W,0.657; 33,YAR002W,0.641; 672,YER009W,0.624; 557,YDR395W,0.577; 2279,YOR185C,0.574;

1692,YLR293C,0.569; 77,YBL079W,0.453; 1533,YKR092C,0.452; 720,YER105C,0.450; 202,YBR267W,0.432; 1528,YKR082W,0.384

Module 59, Branch 112, skewness score: 0.727063

T: 50,MATA1,0.429

T*: 105,SWI5,-0.431; 69,PUT3,-0.348; 2,ACE2,-0.346; 14,CHA4,-0.330; 92,SOK2,-0.276; 113,UME6,-0.258; 73,REB1,-0.236; 80,ROX1,-0.229; 100,STP1,-0.228

gene: 285,YDL014W,0.746; 2421,YPL157W,0.708

Module 60, Branch 112, skewness score: 0.524394

T: 61,NDD1,0.207; 10,AZF1,0.359

T*: 73,REB1,-0.196; 7,ARO80,-0.193; 101,STP4,-0.189; 48,LEU3,-0.188; 90,SNF1,-0.185; 44,INO2,-0.185; 1,ABF1,-0.184; 115,XBP1,-0.183; 26,GAL4,-0.183; 96,STB2,-0.182; 114,URC2,-0.178; 43,IME1,-0.172; 37,HAP1,-0.171; 87,SKN7,-0.169; 113,UME6,-0.150; 100,STP1,-0.150; 105,SWI5,-0.147; 9,ASH1,-0.144; 86,SIP4,-0.128; 103,SUT1,-0.122; 62,NRG1,-0.122; 97,STB4,-0.119; 123,YOX1,-0.119; 98,STB5,-0.118; 64,PDR1,-0.114; 102,SUM1,-0.112; 80,ROX1,-0.108; 2,ACE2,-0.107; 107,TBF1,-0.105; 63,OPI1,-0.103; 59,MSN2,-0.103; 126,ATF1,-0.103; 31,GCR1,-0.102; 82,RPN4,-0.101; 91,SNT2,-0.100

gene: 529,YDR335W,0.550; 883,YGL172W,0.544; 2249,YOR098C,0.535; 1358,YJR042W,0.532; 1425,YKL057C,0.520; 1946,YMR308C,0.487; 1268,YJL041W,0.467; 1874,YMR153W,0.459; 785,YFR002W,0.458; 1798,YML103C,0.453; 1866,YMR129W,0.447; 324,YDL088C,0.415; 1528,YKR082W,0.379; 332,YDL116W,0.370; 720,YER105C,0.356; 77,YBL079W,0.351; 1702,YLR335W,0.332; 971,YGR119C,0.326; 96,YBR017C,0.280; 846,YGL100W,0.280

Module 62, Branch 20, skewness score: 0.580687

T: 6,ARG81,0.408; 30,GCN4,0.442; 83,RTG3,0.499

T*: 24,FKH1,-0.163; 25,FKH2,-0.153; 77,RLM1,-0.126; 93,SPT2,-0.124; 118,YAP5,-0.089

gene: 2160,YOL058W,0.825; 711,YER090W,0.799; 701,YER069W,0.772; 707,YER086W,0.738; 1292,YJL088W,0.728; 193,YBR248C,0.661; 194,YBR249C,0.656; 409,YDR035W,0.649; 2321,YOR303W,0.631;

858,YGL125W,0.622; 1853,YMR096W,0.582; 2201,YOL140W,0.567; 2255,YOR108W,0.563; 2002,YNL104C,0.560; 446,YDR127W,0.549; 1485,YKL211C,0.521; 2129,YNR050C,0.498; 2261,YOR130C,0.495; 2437,YPL188W,0.495; 368,YDL198C,0.490; 2284,YOR202W,0.487; 539,YDR354W,0.480; 898,YGL202W,0.477; 2552,YPR185W,0.472; 890,YGL184C,0.467; 1334,YJL200C,0.455; 143,YBR115C,0.437
