# Supplementary material for: Evolution of transcriptional networks in yeast: alternative teams of transcriptional factors for different species
Source: BMC Genomics. 2016 Nov 11;17(Suppl 10):826. doi: 10.1186/s12864-016-3102-7 (PMC5123246; doi:10.1186/s12864-016-3102-7)
Supplement: Supplementary file 6 — Supplementary material overview. We give an overview of the supplementary material and methods provided in this paper. (PDF 74 kb) [file 12864_2016_3102_MOESM6_ESM.pdf]

Supplementary Materials Overview for Evolution of transcriptional networks in yeast: alternative teams of transcriptional factors for different species

The work presented in this paper relies on a database of the binding probabilities of 126 transcription factors to 2557 genes shared by 23 species; a $126 \times 2557 \times 23$ matrix (stored in `binding_probability_database.txt`). In the few cases where a gene did not exist in a given species, and similarly for transcription factors, the corresponding entries are set to 0.0000. In the cases where a species had multiple copies of the same gene, one was selected at random. The supplementary materials include a text version of the database of binding probabilities. In this file, the 2557 probabilities associated to a given transcription factor and species are separated by commas, the 126 groups of comma separated binding probabilities associated to a given species are separated by semicolons, and the 23 groups of semicolon separated binding probabilities are separated by colons, as follows:

```
P(g=1,t=1,s=1),...,P(g=2557,t=1,s=1);...;P(g=1,t=126,s=1),...,P(g=2557,t=126,s=1)
:...:
P(g=1,t=1,s=23),...,P(g=2557,t=1,s=1);...;P(g=1,t=126,s=23),...,P(g=2557,t=126,s=23)
```

This is essentially the format for .csv files, with an extra dimension. As an example, in Python one could print the binding probability corresponding to the first species, the second transcription factor, and the third gene:

```
with open("regulatory_database.txt", "r") as f:
  print f.read().split(":")[0].split(";")[1].split(",")[2]
```

Recalling that Python indexes from zero.

We have also included the file `species_names.txt`that contain the scientific names for the 23 species (e.g. Saccharomyces cervisiae).Additionally, we included a document describing the gene modules (`Supplementary_all_genes_modules.docx`).

In the interest of providing a complete picture of our results, we have included a document that gives, for each module, the top scoring branch and the associated rewiring block (`Supplement_Main_Rewiring_Blocks_skew.docx`). This

document also includes the names of the genes and transcription factors involved and the overall skew score. Finally, we have included a Supplementary Methods document which details the methods used to generate the binding probabilities (`Supplement_method_TF_binding_probabilities.pdf`).
